# Metabolic Remodeling in Skeletal Muscle Atrophy as a Therapeutic Target

**DOI:** 10.3390/metabo11080517

**Published:** 2021-08-05

**Authors:** Alessandra Renzini, Carles Sánchez Riera, Isidora Minic, Chiara D’Ercole, Biliana Lozanoska-Ochser, Alessia Cedola, Giuseppe Gigli, Viviana Moresi, Luca Madaro

**Affiliations:** 1 Unit of Histology and Medical Embryology, Department of Anatomy, Histology, Forensic Medicine and Orthopaedics, Sapienza University of Rome, 00185 Rome, Italy; alessandra.renzini@uniroma1.it (A.R.); carles.sanchezr@gmail.com (C.S.R.); minic.1916003@studenti.uniroma1.it (I.M.); c.dercole@uniroma1.it (C.D.); biliana.lozanoska-ochser@uniroma1.it (B.L.-O.); luca.madaro@uniroma1.it (L.M.); 2 Institute of Nanotechnology, c/o Dipartimento di Fisica, National Research Council (CNR-NANOTEC), Sapienza University of Rome, 00185 Rome, Italy; alessia.cedola@cnr.it; 3 Institute of Nanotechnology, c/o Campus Ecotekne, National Research Council (CNR-NANOTEC), Monteroni, 73100 Lecce, Italy; giuseppe.gigli@cnr.it

**Keywords:** skeletal muscle metabolism, muscle wasting, physical exercise, diet, epigenetics

## Abstract

Skeletal muscle is a highly responsive tissue, able to remodel its size and metabolism in response to external demand. Muscle fibers can vary from fast glycolytic to slow oxidative, and their frequency in a specific muscle is tightly regulated by fiber maturation, innervation, or external causes. Atrophic conditions, including aging, amyotrophic lateral sclerosis, and cancer-induced cachexia, differ in the causative factors and molecular signaling leading to muscle wasting; nevertheless, all of these conditions are characterized by metabolic remodeling, which contributes to the pathological progression of muscle atrophy. Here, we discuss how changes in muscle metabolism can be used as a therapeutic target and review the evidence in support of nutritional interventions and/or physical exercise as tools for counteracting muscle wasting in atrophic conditions.

## 1. Introduction

Skeletal muscle is composed of heterogenous fibers with different metabolic profiles and functional properties. Muscle fibers are broadly categorized into two groups: the slow-twitch or type 1 fibers and the fast-twitch or type 2 fibers. Because of the differential expression of myosin heavy chains, fast-twitch myofibers are further subdivided into 2A, 2X, and 2B types, and into 1/2A, 2A/2X, and 2X/2B types based on hybrid myosin heavy chain expression [1]. This classification also reflects differences in mitochondrial content and ATP consumption [1,2]. Indeed, type 1 and 2A fibers are primarily oxidative, while type 2X and 2B fibers mainly rely on glycolytic metabolism [1,2]. In addition to metabolism and myosin heavy chain expression, numerous other factors contribute to fiber-type classification, including the expression of specific components of the sarcomere contractile machinery [1,2], the use of alternative splicing for some structural proteins [3,4], or the expression of fiber-specific micro-RNAs [5]. Ultimately, the orchestrated regulation of fiber-type-specific biochemical and physiological features results in unique and specific functional properties for each fiber type. In mammals, a single muscle group is generally formed by multiple fiber types, and different proportions of fiber types lead to diverse muscle groups [1,2]. For instance, a soleus muscle is mainly composed by type 1 slow fibers while a triceps one is predominantly type 2 [6]. Nevertheless, muscle fibers can remodel their phenotypes in response to external demand. Depending on the stimuli, muscle atrophy may affect specific fiber types, involving predominantly slow type I or fast type II muscle fibers [7]. Indeed, muscle fiber subtypes respond differently to distinct signaling pathways. In general, fast-twitch glycolytic fibers are more prone to be affected by atrophic conditions than slow-twitch oxidative fibers [8].

Muscle atrophy is often associated with metabolic reprogramming, which contributes to phenotypical changes and protein degradation. Conditions known to induce changes in muscle metabolism and initiate muscle wasting include aging, denervation, or neurodegenerative diseases, such as amyotrophic lateral sclerosis (ALS), and syndromes, such as cancer cachexia. Compromised muscle homeostasis, due to a decrease in protein synthesis, and perturbed proteolytic pathways are features shared by all of the above conditions; however, differences in muscle metabolic reprogramming have been reported, dependent on different stimuli [7]. For instance, decreased usage of skeletal muscle causes an adaptive metabolic remodeling characterized by lower capacity for fat oxidation and switch to glycolysis as the main fuel supply, associated with a transition from slow to fast myosin fiber types [9]. Instead, increased fatty acid oxidation and glucose intolerance, associated with a shift in fast-to-slow muscle fiber type composition [10], have been reported in a mouse model of ALS [11].

As an active part of the muscle wasting process, metabolic remodeling represents an attractive therapeutic target for the re-establishment of muscle homeostasis. Thus, the use of thyroid hormone prevents fasting-induced skeletal muscle atrophy by inducing metabolic adaptation, without affecting muscle synthesis or degradation [12]. Moreover, physical exercise or specific nutrient intake can counteract skeletal muscle atrophy in different pathophysiological conditions [13,14,15]. In this review, we will give an overview of the involvement of metabolic remodeling in muscle pathophysiology upon different stimuli, as well as the signaling pathways underlying compromised muscle homeostasis. We will discuss how physical exercise or nutritional support can counteract muscle wasting, proposing them as nonpharmacological approaches to restore muscle homeostasis, even in disease contexts.

## 2. Skeletal Muscle Metabolic Reprogramming during Aging

**Figure 1:**
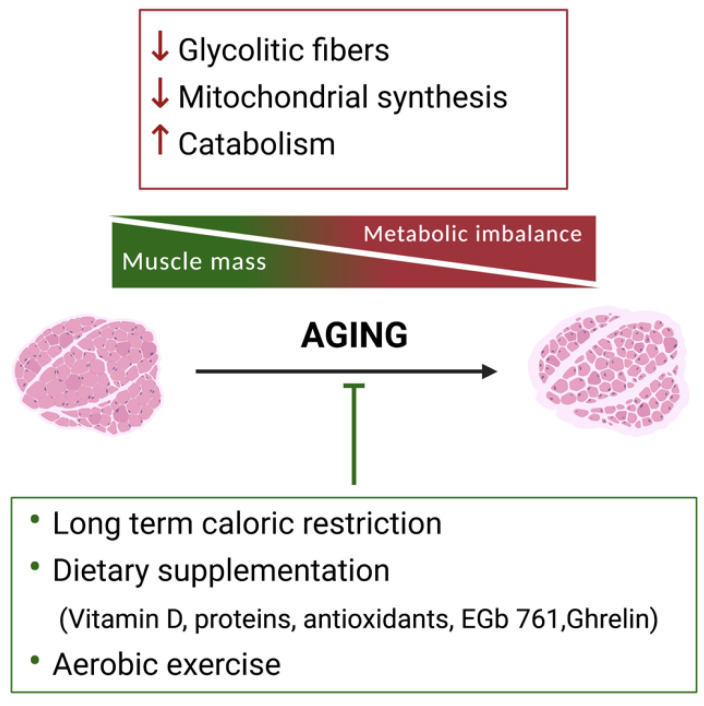
Metabolic reprogramming of skeletal muscle during aging and non-pharmacological approaches to counteract sarcopenia. Skeletal muscle metabolic remodeling during aging is associated with a reduction in the number of glycolytic fibers, reduced mitochondrial synthesis, and increased catabolism (red box). Decreased anaerobic glycolysis and impaired mitochondrial activity result in elevated protein catabolism and loss of muscle maintenance. Sarcopenic muscle shows reduced muscle fiber size (atrophy) and number (hypoplasia), and is accompanied by fat infiltration and connective tissue deposition. Among the main non-pharmacological approaches for the prevention of muscle mass loss during aging are long-term caloric restriction, dietary supplementation, and aerobic exercise (green box).

Muscle is a highly diverse and adaptive tissue [16]. However, during aging, changes in metabolism and accumulation of reactive oxygen species (ROS) are associated with loss of muscle mass and decline in muscle function rather than being part of an adaptive response [17] (Figure 1). Loss of muscle mass is proportional to the passage of time, increasing from 1–2% around 50 years of age, to 40% in people older than 80 years who are considered sarcopenic [18,19]. Related to muscle fiber loss, there is a decline in muscle functions, such as temperature regulation [20]. Moreover, there is an imbalance between anabolic and catabolic processes in skeletal muscle, with a decrease in anabolic agents such as insulin-like growth factor (IGF-1), testosterone, and growth hormone (GH), and an increase in catabolic signals such as interleukin 6 (IL-6) and tumor necrosis factor-alpha (TNF-α) [21]. Indeed, sarcopenia is increasingly recognized as an inflammatory state driven by cytokines and oxidative stress [22] that negatively impacts on muscle protein turnover, by decreasing protein synthesis [23].

While in the early stages of development, cell energy (ATP) and oxidative phosphorylation are generated through the oxidation of carbon sources such as lactate, pyruvate, amino acids, and fatty acids [24,25,26]; during aging, muscles are subjected to metabolic changes. For instance, mitochondrial activity impairment and changes in energy metabolism are critical to the development of sarcopenia [27]. Mitochondria in skeletal muscles of the elderly display impaired respiration, a decreased activity of mitochondrial enzymes, and reduced mitochondrial biogenesis and content [28,29]. Thus, oxidative muscles, which are endowed with greater mitochondrial content, are able to preserve their oxidative capacity during aging-associated changes, unlike glycolytic muscles, which are more prone to becoming impaired [30].

Muscle loss in sarcopenia results from a fiber type-specific loss of muscle mass (muscle atrophy) and reduced number of specific fiber types (hypoplasia). Numerous studies have shown that during aging, type II fibers are more susceptible to atrophy compared to type I fibers [31,32]. Moreover, type IIB fibers undergo atrophy to a greater extent compared to type IIA fibers, irrespective of gender [33,34]. As for the hypoplasia, up to the age of 80 years, type II muscle fibers are the main fiber type lost in postural muscles [32], leading to an increase in the ratio of type I/II fibers; whereas, after 80 years of age, there is a similar decline in the number of type I and II fibers [35,36], which ultimately leads to a similar type I/II fiber ratio after the age of 85 years [37]. In addition, aging is associated with increased heterogeneity in MHC isoforms due to the preferential loss of fast motoneurons [38], which is thought to induce a shift towards the slower phenotype. This event, combined with the disuse-induced muscle atrophy, leads to the expression of fast MHC isoforms and physical activity able to reverse the shift towards a slower phenotype [39]. Thus, a distinctive MHC distribution in aged muscles does not exist, being the result of age-dependent neurodegenerative processes and physical activity status, which differ across individuals.

Another consequence of muscle mass loss is the increased susceptibility to injury and reduced capacity to regenerate [40]. This reduced capacity to regenerate has been linked to the loss of muscle satellite cells (MuSCs), the muscle stem cells, and metabolic reprogramming [41]. Available evidence suggests that the metabolite balance of both stem and differentiated cells can directly influence the epigenetic landscape through post-translational modifications of histone proteins, DNA sequence, and transcription factors [42,43]. Therefore, changes in metabolism may regulate many of the important cell fate decisions made by stem cells [44,45]. The impaired regeneration capacity of aged skeletal muscle has also been attributed to a decrease in the number of MuSCs, which after several cycles of regeneration/degeneration become exhausted and decline [46]. This decline in MuSC activity is accompanied by changes in the stem cell niche and/or cell-autonomous mechanisms such as oxidative damage [47]. Indeed, the MuSC transcriptome during aging is extensively reprogrammed, switching from genes involved in homeostasis to genes involved in tissue-specific stresses, such as DNA damage or inefficient autophagy [48].

Skeletal muscle metabolic remodeling during aging is associated with a reduction in the number of glycolytic fibers, reduced mitochondrial synthesis, and increased catabolism (red box). Decreased anaerobic glycolysis and impaired mitochondrial activity result in elevated protein catabolism and loss of muscle maintenance. Sarcopenic muscle shows reduced muscle fiber size (atrophy) and number (hypoplasia), and is accompanied by fat infiltration and connective tissue deposition. Among the main non-pharmacological approaches for the prevention of muscle mass loss during aging are long-term caloric restriction, dietary supplementation, and aerobic exercise (green box).

### 2.1. The Use of Specific Nutrients to Counteract Sarcopenia

An important aspect of aging is that the elderly consume less food compared with the young: they are less hungry and thirsty, eat smaller meals, and snack less [49]. Low food intake and monotonous diets put older people at risk of having malnutrition. Thus, in a vicious cycle, declining muscle strength and physical capability in older age may increase the risk of malnutrition, whilst malnutrition may contribute to further decline in physical capability [50]. Nonetheless, it is important to differentiate between malnutrition with deleterious consequences for skeletal muscle, and caloric restriction, which is a dietary regimen that reduces food intake without incurring malnutrition. Long-term caloric restriction has been demonstrated to significantly prevent the age-associated rewiring of the transcriptome [48]. However, in spite of its beneficial effects, caloric restriction is not enough to counteract aging, a phenomenon that is characterized by slow and progressive accumulation of epigenetic errors [51].

The nutrients that have been most consistently used to fight sarcopenia and frailty in older adults are vitamin D, proteins, and a number of antioxidant nutrients, that include carotenoids, selenium, and vitamins E and C [52]. Vitamin D has a genomic impact on skeletal muscle through the interference of the 1.25-VDR-RXR (retinoid receptor) heterodimer at certain nuclear receptors that influence gene transcription. Moreover, vitamin D has a non-genomic effect through a quick activation of intracellular signal transduction of 1.25 (OH) D to its non-nuclear receptors [53]. Interestingly, low serum levels of vitamin D are associated with an increased risk of sarcopenia in elderly adults [54]. Consistently, studies have reported that dietary supplementation with vitamin D improves muscle mass, function, and quality of life in sarcopenic elderly [55,56].

Another important nutrient for the elderly is, of course, protein. Dietary protein provides amino acids that are needed for the synthesis of muscle sarcomeric proteins. Importantly, absorbed amino acids have a stimulatory effect on muscle protein synthesis after feeding [57]. A blunted essential amino acid (EAA) response of muscle protein synthesis occurs in aged humans confined to bed rest, and it involves reduced mTORC1 signaling and amino acid transporter protein content. Supplementation with branched-chain amino acids (BCAA), and in particular leucine, plays an important role in the stimulation of postprandial muscle protein synthesis [58]. Nevertheless, protein turnover still decreases, even after supplementation with both types of amino acids, with continued loss of muscle mass.

Exploring possible treatments, it is necessary to mention EGb 761, a Ginkgo biloba extract, which has been shown to be an effective treatment in chronic age-dependent neurological disorders, by reversing the age-related metabolic shift from lipids to glucose utilization, promoting myogenesis, and restoring a more youthful gene expression pattern in sarcopenic rats [59].

Another possible treatment is represented by ghrelin. Ghrelin is a circulating peptidyl hormone mainly produced by the stomach, which, by acting on the hypothalamus and the pituitary, induces a strong release of GH and stimulates food intake and adiposity [60]. Elderly subjects have diminished levels of plasma ghrelin and these lower levels correlate with a decline in nutritional status [61]. Ghrelin exerts a strong and direct anti-atrophic activity on skeletal muscle and promotes skeletal muscle regeneration, showing anti-catabolic and anti-inflammatory effects, and leading to the inhibition of muscle protein catabolism [62,63]. Despite promising results in preclinical studies, further investigations are needed to clarify the benefits of the aforementioned specific nutrient supplementations on sarcopenia.

### 2.2. Exercise to Counteract Sarcopenia

Physical exercise, alongside a balanced diet, can contribute towards the prevention of muscle mass decline by increasing protein synthesis [17,64,65], inhibiting protein degradation, and stimulating muscle anabolism [65] (Figure 1). The cellular processes regulating the anabolic responses to exercise are more complex than those driven by nutrition alone. Thus, resistance-type exercise triggers multiple intramuscular signaling networks associated with cellular, biochemical, mechanical, and metabolic stress [64]. For example, while some authors have proposed a canonical pathway of regulation for mTORC1 via IGF1–PI3KAkt/PKB–mTOR, recent evidence points toward the existence of muscle intrinsic mechanosensitive signaling pathways that activate mTORC1 post exercise, such as the production of the lipid second messenger, phosphatidic acid (PA)/phospholipase D (PLD) [66,67], and adhesion proteins such as focal adhesion kinase (FAK) [68].

Skeletal muscle adaptation to aerobic exercise involves increased oxygen extraction and utilization [69], principally governed by mitochondrial capacity and function, which leads to increased biogenesis after 24 h of exercise [70]. As a proof of principle, lifelong aerobic exercise effectively reduces the biomarkers associated with sarcopenia in mice, preventing the aging-induced impairment of catabolic pathways and mitochondrial dysfunction, thereby counteracting and delaying aging-induced skeletal muscle atrophy [71]. However, similar to nutritional intake, the basal mitochondrial fractional synthesis rates decrease with aging [72], rendering the skeletal muscle adaptation to exercise less effective. Additional clinical research is needed to address the effectiveness of combined resistance and aerobic exercise protocols to counteract skeletal muscle sarcopenia.

## 3. Metabolic Alteration in Neurodegenerative Diseases

Neurodegenerative diseases, including amyotrophic lateral sclerosis (ALS), Alzheimer’s disease (AD), Parkinson’s disease (PD), and Huntington’s disease (HD), are characterized by progressive degeneration of specific neurons. Once neurodegeneration begins, the inexorable progression can only be slowed but not prevented. Protein aggregation, oxidative stress, and mitochondrial dysfunction are among some of the metabolic alterations observed in neurodegenerative diseases [73]. Whether these metabolic abnormalities play a direct role in the onset of these diseases or arise as a consequence of genetic and epigenetic factors is a matter of debate and remains to be elucidated.

### 3.1. Skeletal Muscle Metabolic Alterations in ALS

**Figure 2:**
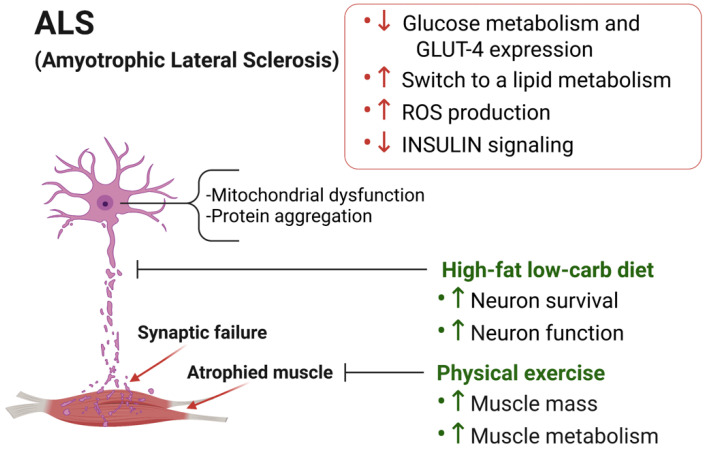
Skeletal muscle metabolic reprogramming and proposed approaches to counteract disease progression. Among the metabolic pathways affected in ALS muscle are glucose and lipid metabolism, reactive oxygen species (ROS) production and insulin signaling (red box). Reduced glucose transport and uptake, increased lipid metabolism, and ROS-mediated oxidative stress result in metabolic dysfunction. A high-fat-low-carb diet and physical exercise have been shown to have protective effects on neurons and skeletal muscles in ALS.

ALS is a neurodegenerative disease affecting the upper and lower motor neurons, resulting in muscle weakness, denervation, and eventual death [74]. Multiple mechanisms, including neuron excitability, glutamate toxicity, and protein aggregation, underlie the pathology of ALS. Cellular metabolic defects arise early and contribute to the clinical manifestation of the disease (Figure 2). Indeed, weight loss, dyslipidemia, and hypermetabolism are some of the metabolic alterations seen in affected patients (reviewed in [75,76]) and correlate with a worsening of the pathology. Despite this, the early events underlying the defective muscle metabolism in ALS as well as the major molecular mechanisms that cause ALS-associated hypermetabolism are still unknown. Indeed, it remains to be determined whether hypermetabolism is an epiphenomenon that might not be causally related to ALS progression, but rather, might simply be a result of muscle denervation.

Similar to human patients, the SOD1^G93A^ mouse model of ALS displays an increased energy expenditure as well as a decline in fat mass during the course of the disease [77,78]. Furthermore, the circulating level of fatty acids (NEFA) is not increased, suggesting that lipids that enter the circulation are rapidly utilized [77] (Figure 2). Since skeletal muscle in resting condition accounts for 20–30% of the total energy expenditure, it is reasonable to assert that alterations in the metabolic rate of this tissue may be crucial for disease progression.

It is interesting to note that denervation of glycolytic fibers is an early event in ALS muscles, suggesting that the metabolic signature of muscle fibers may be an important determinant of disease onset. As described for the mouse model of acute denervation, a reduction in glucose metabolism and GLUT4 expression and a switch to a lipid metabolism have been observed in asymptomatic ALS muscles [11,78,79,80]. This correlates with another early event in ALS, the increase in ROS as a consequence of elevated β-oxidation. In turn, ROS-mediated oxidative stress negatively impacts the insulin signaling cascade and alters the transcription of the glucose transporters responsible for basal and insulin-stimulated glucose uptake [81].

Although the loss of glycolytic fibers is a consequence of the degeneration of fast fatigable motor neuron contact that leads to a shift in muscle fiber metabolism, a cell autonomous effect has also been observed. Indeed, the dependency on fat oxidation is increased in ALS muscle cells, compared to healthy ones, suggesting a cell autonomous defect in energy metabolism [77]. In line with this observation, muscle-specific expression of mutated SOD1^G93A^ causes mitochondria abnormalities and dysfunction in vivo [82,83], parallel to neurovascular alterations, observed through X-ray-Phase-Contrast-Tomography (XPCT), at different stages of the disease [84]. XPCT demonstrated the ability to detect neuron alterations even in a pre-symptomatic state in SOD1^G93A^ mice. These mice display a shift from glycolytic toward slow oxidative fiber metabolism composition and a reduction in GLUT4 expression and glucose metabolism, resulting in muscle glycogen accumulation [83] (Figure 2).

Counteracting this event may represent a potential pharmacological strategy to delay disease progression. Indeed, fatty acid oxidation inhibition and switching to the use of glucose as fuel delay the onset and ameliorate disease progression [11,85].

It should be emphasized that metabolic alterations may differ during different stages of the disease. In addition, the different tissues involved (i.e., neurons, muscle, glial cells etc.) have different energy demands and substrates. This together with the heterogeneity of the pathology in different patients represents an area of research that deserves further investigation.

Among the metabolic pathways affected in ALS muscle are glucose and lipid metabolism, reactive oxygen species (ROS) production, and insulin signaling (red box). Reduced glucose transport and uptake, increased lipid metabolism, and ROS-mediated oxidative stress result in metabolic dysfunction. A high-fat, low-carb diet and physical exercise have been shown to have protective effects on neurons and skeletal muscles in ALS.

### 3.2. Dietary Intervention to Counteract ALS Progression

An increased body mass index (BMI) corresponds to an improvement in the lifespan of ALS patients, while a decreased BMI may increase disease severity [86,87,88,89], suggesting that dietary interventions can potentially alter the course of the disease. It is well known that lipolysis and fatty acid mobilization are a consequence of increased muscle energy requirements [90]; therefore, it is likely that the increased mobilization of lipids in an ALS mouse model occurs as a means to sustain a metabolic requirement in peripheral skeletal muscle [91]. Indeed, fatty acid metabolism provides more ATP compared to glucose metabolism [92], and Steyn and colleagues found that slower clinical decline in ALS patients was associated with higher fatty oxidation metabolism in myotubes [93]. Furthermore, a pilot study showed that a high caloric diet based on high carbohydrate content was well tolerated by ALS patients, and was associated with reduced serious adverse events [94].

This discovery is supported by a recent work showing that a high-fat diet is not only able to slow ALS progression but also to reduce the risk of developing ALS pathology [95,96], and a high level of circulating cholesterol and triglycerides is associated with prolonged survival of ALS patients [97]. Consistently, a high-fat diet improves motor function and increases survival of both TDP-43^A315T^ and SOD1^G93A^ ALS mouse models [98,99], supporting the idea that dietary supplementation may support hypermetabolic demand. Conversely, caloric restriction significantly reduced the survival of SOD1^G93A^ mice [100,101]. Mitochondria dysfunction and decreased activity of complex I are also associated with ALS pathology [102,103]. This observation suggests that a ketogenic diet (a high-fat, low-carb diet) may be optimal to support both hypermetabolic demand and to restore the function of complex I, promoting ATP synthesis. Indeed, a ketogenic diet prevents neuron loss and increases the lifespan of SOD1^G93A^ mice [104]. Similarly, a diet supplemented with caprylic triglyceride, as a source of ketone bodies, improves motor function in the same ALS mouse model [105].

In summary, available epidemiologic evidence supports the notion that malnutrition may contribute to ALS progression both in humans and in mice; conversely, increased fat and cholesterol intake might reduce the risk of ALS and the rate of disease progression (Figure 2). Based on the encouraging data from animal studies, there is now a great interest in pursuing dietary interventions for ALS. However, more clinical research is needed to explore the potential of the high-fat or ketogenic diet in the treatment of ALS.

### 3.3. Physical Exercise in ALS Progression

In addition to dietary interventions, physical exercise may also be an important modulator of muscle metabolism (Figure 2). Indeed, while low-intensity exercise induces a shift toward oxidative lipid metabolism, high-intensity exercise turns on the glycolytic metabolic pathway [106,107]. Moreover, exercise can promote the clearance of damaged mitochondria through the activation of the catabolic machinery [108]. Despite this evidence, the use of exercise as an adjunct therapeutic approach is still controversial. Indeed, epidemiologic data have revealed a higher incidence of ALS in subjects performing physical activities and athletes [109,110,111,112], while other studies have shown opposite results [113,114,115]. Without going into the merits of whether or not physical activity can facilitate the onset of ALS, we wish to explore the potential of exercise as a therapeutic intervention designed to slow disease progression.

Several clinical trials have demonstrated the benefits of exercise in ameliorating disease symptoms. Moderate exercise (i.e., 15 min performed twice daily) delays the deterioration of motor function and attenuates the decline in muscle strength [116,117,118]. Unfortunately, this beneficial effect is lost during disease progression [117]. While moderate exercise has been found to increase the lifespan of a SOD1^G93A^ mouse model of ALS [119], high-intensity exercise has no effect or is detrimental to the survival of these mice [120]. The quality of performed exercise seems to be critical for the beneficial effect observed in ALS. Thus, activation of the fast motor unit and the switching to glycolytic muscle metabolism exert beneficial effects in a mouse model of ALS [79]. However, in humans, research data are less clear. Overall, it seems that the best option to prevent the onset of fatigue and see improvements in ALS patients is an exercise of moderate intensity and not very high frequency, combining strength and aerobic endurance [121].

With the aim to clarify some of this discrepancy, Gerber et al. examined the effect of different running intensities on the survival of SOD1^G93A^ mice [120]. In contrast to previous studies, the authors used an appropriate control mouse, sedentary, and mice placed in a “sedentary treadmill” (treadmill powered off). Exercise did not improve mice survival at all the intensities analyzed (5, 10, and 21 cm/s, 15 min/day, 5 days/week). Nevertheless, increasing the intensity of exercise promoted the maintenance of body weight by increasing muscle mass [120].

Although useful, the results of these animal studies are difficult to reproduce in humans where the diagnosis of the disease is often made when the neurological damage is already significant. A weak or denervated muscle is more susceptible to overwork damage because it is already functioning close to its maximal limits. The hope is that in the future we will be able to design an appropriate exercise protocol, perhaps associated with an ideal diet, in order to maximize the chance of slowing down the pathology.

## 4. Skeletal Muscle Metabolism in Cancer Cachexia

**Figure 3:**
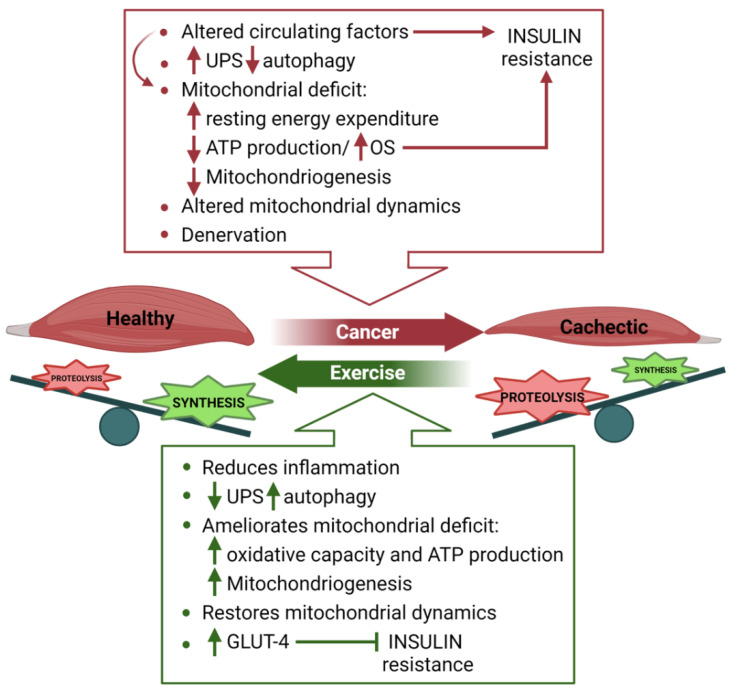
Metabolic reprogramming in cancer-induced cachexia and beneficial effects of exercise. In the red box are summarized the multiple pro-cachectic effects mediated by cancer. Impaired autophagy, energy expenditure, and denervation result in unbalanced protein homeostasis, overall leading to muscle wasting. In the green box are listed the anti-cachectic protective effects induced by exercise. Increased anti-inflammatory cytokines and suppression of the catabolic pathways lead to increased muscle mass and improved metabolism.

Cancer cachexia is a multifactorial syndrome characterized by severe skeletal muscle wasting with or without adipose tissue loss, that develops irrespective of the given nutritional support [122]. It is a common feature of advanced cancer, caused by a combination of tumor- and host-derived factors, reduced food intake, and abnormal metabolism [123] (Figure 3).

Functional muscle mass is maintained by a dynamic balance between protein synthesis and degradation; a decrease in synthesis or an excessive degradation results in muscle wasting [124]. During tumor progression, the systemic network of anabolic and catabolic factors which regulates the balance between muscle synthesis and degradation is heavily compromised [125,126] (Figure 3). A decrease in circulating anabolic factors, such as insulin growth factor-1 (IGF-1) [127], leads to defective glucose handling and insulin resistance in tumor-bearing mice or cancer patients [128,129], overall contributing to muscle loss. A contemporaneous increase in circulating proinflammatory cytokines, which promote muscle catabolism, has been described in cachectic patients [130]. Moreover, tumor-derived extracellular vesicles (EVs) that promote myofiber death have been recently reported [131], further proving that a crosstalk between tumor and skeletal muscle contributes to the progression of cancer cachexia. Interestingly, besides signal transduction and cell communication, cell metabolism is one of the most common biological processes affected by tumor-derived EVs in cancer patients [132]. Additional studies will be necessary to determine the specific impact of EVs in mediating the crosstalk between tumor and skeletal muscle.

Recently, the involvement of neurogenic muscle atrophy was reported in a murine model of cancer cachexia [133], which may participate in muscle catabolism or metabolic reprogramming. Given that there is some controversy regarding this topic [134], further studies are needed to better define the involvement of compromised neuronal muscular junctions and muscle innervation in the progression of cancer cachexia.

Mitochondrial dysfunction associated with reduced ATP production [135,136,137,138] and elevated oxidative stress has been described in cachectic muscles [139,140,141,142]. Interestingly, mitochondrial dysfunction occurs before the onset of muscle wasting in tumor-bearing mice, suggesting a causative role in the activation of the proteolytic pathways [143]. In addition to the mitochondrial functional deficit, decreased mitochondriogenesis and altered mitochondria dynamics, probably due to high levels of circulating proinflammatory cytokines, contribute to altered muscle metabolism in cancer cachexia [144]. High resting energy expenditure is also typical of cancer patients [145], probably due to the increased expression of the uncoupling proteins UCP2 and UCP3 in cachectic muscles [146]. Dysfunctional mitochondria and compromised oxidative metabolism may, in turn, contribute to muscle insulin resistance and reduced protein metabolism [147,148,149].

While significant alteration in muscle metabolism has been extensively reported, metabolic reprogramming is not related to changes in fiber type composition, in cachectic patients [150,151,152] or in cancer cachexia murine models [153]. Instead, cachectic muscles show myosteatosis, i.e., accumulation of intramyocellular lipid droplets, both in humans and in rodents [154,155], which is associated with shorter survival [156]. Although abnormal expression of miRNAs [157], single nucleotide polymorphism [158], or alternatively spliced genes [159] associated with lipid metabolism have been reported, the mechanisms underpinning myosteatosis have not been fully elucidated.

In the red box are summarized the multiple pro-cachectic effects mediated by cancer. Impaired autophagy, energy expenditure, and denervation compromise protein homeostasis, leading to muscle wasting. In the green box are listed the anti-cachectic protective effects induced by exercise. Increased anti-inflammatory cytokines and suppression of the catabolic pathways lead to increased muscle mass and improved metabolism.

### 4.1. Nutritional Support to Counteract Cancer Cachexia

Muscle wasting correlates with poor prognosis in cancer patients, increasing morbidity and reducing both tolerance and responsiveness to treatments, ultimately accounting for up to 30% of deaths associated with cancer [160]. Importantly, preserving muscle mass extends survival in animal models of cancer cachexia [161,162]. Thus, interventions aimed to preserve functional muscle mass offer considerable potential in combination with anti-cancer regimens to enhance patient outcomes. Although nutritional support per se does not efficiently reverse the cachectic syndrome, it must be considered as an important component of a multi-targeted approach. To this purpose, nutritional therapies are under investigation as combined therapy, supporting a multimodal approach to counteract cachexia syndrome [163]. Anorexia and inadequate food intake occur in advanced cancer patients; therefore, dietary counseling and supplements are often suggested [164,165].

To improve muscle anabolism and help fight muscle wasting in cancer cachexia, the ketogenic diet characterized by increased protein intake has been proposed as a promising adjuvant treatment for cancer patients [166]. Although the optimal amount of proteins for muscle maintenance or gain has not yet been defined [167], a number of studies have suggested a dietary protein intake of >1.5 g/kg/day as a combined approach to counteract muscle wasting in cancer patients [168,169]. Interestingly, red and processed meat consumption is associated with higher risk of developing multiple cancers and with increased risk of overall cancer mortality [170], pointing to the importance of defining the optimal quantity, composition, and timing of protein intake.

Numerous mechanisms underlie the anti-tumor effects of ketogenic diets, including the reduced intake of glucose, which represents the primary metabolic fuel for many cancers because of the Warburg effect, and the promotion of ketone bodies synthesis, which reduce oxidative stress and exert anti-inflammatory action. Despite numerous studies and clinical trials demonstrating the potential of ketogenic diets [171], short- and long-term side effects of such rigid dietary regimens must be managed by a qualified nutritionist that should tailor the specific diet to individual cancer patients.

In addition to general nutritional strategies, numerous appetite stimulants such as megestrol acetate [172], l-carnitine [173], endocannabinoids [174], and ghrelin [175] have been the subject of recent clinical trials for their potential to increase food intake, thus promoting body weight gain and quality of life in cancer patients [176].

Establishing a net beneficial effect on skeletal muscle driven by nutrients without stimulating tumor growth, or negatively influencing anti-tumor therapy in cancer-related cachexia is a considerable challenge [177]. A number of dietary supplements, including omega-3 polyunsaturated fatty acids (N-_3_ PUFAs), polyphenols from fruits and tea, and vitamin antioxidants are known to exert anti-inflammatory effects in cancer conditions [178,179,180]. N-_3_ PUFAs, mainly sourced from marine fish and fish oil, have been reported to reduce cancer cachexia [181] as well as tumor growth [182,183], in addition to exerting numerous other health benefits in animals and humans [184,185].

Docosahexaenoic acid (DHA) and eicosapentaenoic acid (EPA) are the major effective PUFAs reported to improve body composition, muscle mass, and function [186,187] in cancer patients, and to suppress angiogenesis and cancer growth by multiple mechanisms [178,188]. Several lines of evidence also support a positive correlation between a higher intake of N-_3_ PUFAs and prolonged survival of cancer patients [178,189]. Previous studies have demonstrated a direct effect of N-_3_ PUFAs on muscle growth and hypertrophy. For instance, C2C12 myotubes supplemented with N-_3_ PUFAs show increased intracellular anabolic signaling with a concomitant increase in mitochondrial biogenesis, through enhanced expression of PPAR, PGC-1 α, carnitine palmitoyltransferase 1α and β (CPT1α; CPT1β), TFAM, and NRF genes [190,191]. Further studies confirmed that N-_3_ PUFAs increase muscle anabolic responses in human subjects [192,193,194,195], and reduce the catabolic pathways, lipid mobilization, and glucose consumption in cachectic patients, overall improving the body weight, lean body mass, and quality of life of cancer patients [178,196].

Among the polyphenols, epigallocatechin-3-gallate (EGCG), curcumin, resveratrol, and quercetin have been reported to reduce the catabolic pathways in cachectic muscle, by inhibiting the UPS pathway, whilst inducing IGF-1 anabolic signaling [197]. Moreover, resveratrol and quercetin are known inducers of SIRT1, thereby promoting mitochondrial biogenesis, preserving muscle oxidative capacity and increasing exercise tolerance [198,199,200], in addition to exerting anti-inflammatory, antioxidant, and anticarcinogenic effects through several mechanisms [201].

Several studies have proposed amino acid supplementation to improve cancer-induced muscle wasting by directly acting on muscle cells. In particular, a branched-chain amino acids (BCAAs) (i.e., valine, isoleucine, leucine)-enriched diet has been reported to counteract the negative protein turnover in skeletal muscle, without affecting tumor growth in cancer cachexia [202,203,204,205]. The positive effect of BCAAs on muscle protein turnover is mediated by multiple mechanisms, which remain to be fully clarified [206]. A recent study demonstrated that BCAAs drive increased phosphorylation of mTOR, 70 kDa ribosomal S6 kinase (*p*70^S6k^), and eIF4E-binding protein 1 (4E-BP1), parallel to the dephosphorylation of the eukaryotic initiation factor 2α (eIF2α), overall increasing protein synthesis while reducing protein degradation in MAC16 tumor-bearing mice [205].

A number of mechanisms by which vitamin D interferes with skeletal muscle function have been elucidated in muscle cell lines and in animal models, including modulation of muscle metabolism, by enhancing sensitivity to insulin, and mitochondria biogenesis and function [207,208]. Reduced circulating levels of vitamin D have been associated with impaired glucose metabolism and insulin sensitivity, in addition to a decrease in muscle mass, and performance in several pathologies [209,210,211,212]. In addition, vitamin D deficiency is highly prevalent in advanced cancer-related cachexia patients [213,214], highlighting the importance of vitamin D supplementation in this disease. However, contrasting activities of vitamin D have been reported in muscle cell lines and in tumor-bearing animals [180,213]. For instance, both pro- and anti-atrophic effects have been reported for two vitamin D metabolites on C2C12 myotubes upon addition of cancer cell-conditioned medium [215], discouraging the use of this treatment in vivo for rescuing cancer cachexia. Similarly, tumor-bearing rats treated with vitamin D displayed an impairment in the muscle regenerative program and any beneficial effect in counteracting muscle wasting [216]. In contrast, another study demonstrated vitamin D’s ability to reverse tumor-cell mediated changes in mitochondrial oxygen consumption and proteasomal activity in human skeletal muscle myoblasts [217], thus supporting the use of vitamin D to treat muscle wasting in cachexia. Differences in the experimental design (i.e., tumor-related cytokines in the conditioned medium) may account for the reported discrepancies regarding the effect of vitamin D on muscle wasting.

Overall, numerous studies support the use of dietary supplements to counteract muscle wasting in cancer cachexia; however, the effective doses, long-term effects, and potential side effects need to be further investigated to clearly determine the efficacy of these compounds. Moreover, additional experiments must investigate any potential effects of specific nutrients in conjunction with other pharmacological treatments, such as chemotherapy, on muscle homeostasis in cancer patients.

### 4.2. Exercise to Fight Cancer-Induced Cachexia

Physical exercise has been widely proposed as a promising intervention strategy for the prevention and treatment of cancer-related cachexia (Figure 3). In addition to favoring an increase in anti-inflammatory cytokines (i.e., IL-10, IL-1 receptor antagonist, and soluble TNF receptors-1 and-2) at the expense of pro-inflammatory cytokines, such as TWEAK, IL-1, INF-gamma, and LIF, among others [218,219], exercise negatively affects tumor mass growth and significantly improves muscle metabolism, highlighting the promising beneficial effects of exercise as a multitarget tool in the treatment of cancer cachexia [220,221,222]. Aerobic, resistance, and combined exercise training have been proven to be beneficial for cancer-induced muscle wasting, by modulating muscle mass and metabolism [223].

Exercise counteracts loss of muscle mass and functionality through suppression of catabolic pathways, whilst improving protein synthesis in cancer cachexia. Importantly, the amount of aerobic exercise directly correlates with the lifespan of tumor-bearing mice [162], highlighting the direct involvement of exercise in rescuing cancer-induced cachexia. Several studies have demonstrated exercise-driven modulation of the ubiquitin–proteosome system (UPS) and autophagy. Indeed, aerobic exercise reduces the gene expression level of the E3 ubiquitin ligases MuRF1 and Atrogin-1 in muscles of C26-bearing mice, thereby reducing muscle proteolysis [162,224]. Moreover, cachectic muscles are characterized by an accumulation of autophagic markers as a consequence of autophagic flux overloading [162,225]. Exercise triggers the autophagic flux [226], restoring Beclin-1 levels, including both isoforms of LC3b and p62, and ultimately leads to improved muscle homeostasis in tumor-bearing mice [162,223,224]. Similar results have been obtained administering pharmacological compounds known to trigger autophagy, i.e., AICAR and rapamycin, in tumor-bearing mice [162,227,228]. Moreover, a complete inhibition in the autophagic flux is deleterious in cancer cachexia, as proven by the early death of cachectic mice treated with colchicine [162], and further confirmed by increased muscle wasting promoted by knocking down Beclin-1 in cachectic muscles [229].

The anabolic resistance in cancer-induced muscle wasting seems to be attenuated by resistance exercise training through improvement in mammalian target of rapamycin (mTOR) signaling [230]. Indeed, muscle contraction triggers the expression of IGF-1 [231], which, in turn, activates several intracellular kinases, including phosphatidylinositol 3-kinase (PI3K), that promotes protein kinase B (Akt) activity, thereby promoting protein synthesis via the mTOR and glycogen synthase kinase 3β (GSK3β) kinases, in addition to counteracting protein degradation by suppressing the activation of the Forkhead (FoxO) transcription factors family.

In addition, mTOR and mitochondrial functions are strongly integrated. Indeed, mTOR modulates nuclear-encoded mitochondrial mRNAs and mitochondrial ribosomal protein translation through a 4E-BP1-dependent mechanism, thereby modulating mitochondrial mass, mitochondria biogenesis, and mitophagy [230]. Moreover, resistance exercise triggers the activity of other crucial genes responsible for mitochondrial biogenesis and dynamics, i.e., PGC1-α, NRF-1 and TFAM, overall enhancing mitochondria oxidative capacity and ATP production in different tumor-bearing animal models [224,232,233,234]. While suppressed PGC-1α expression in cachectic muscles correlates to disrupted mitochondrial dynamics and increased muscle wasting [144,235], discordant results have been reported on the effects of PGC-1α upregulation in skeletal muscle in Lewis lung carcinoma (LLC)-bearing mice. Thus, an increase in muscle mass was reported in Pin et al. [236], whilst cancer cachexia was not counteracted in Wang et al. [237] in PGC-1α overexpressing muscles LLC-bearing mice. Both studies reported an increase in tumor mass [236,237], highlighting the cross-talk between muscle and tumor and revealing a possible limitation of increasing the expression of PGC-1α in muscle in cancer cachexia [238]. In addition to improving mitochondrial oxidative capacity, exercise modulates muscle metabolism by enhancing GLUT-4 translocation at the sarcolemma [239], thereby increasing the productivity of the glycolytic pathway and counteracting insulin resistance [239,240,241].

In humans, several clinical trials have shown that physical activity during and after cancer treatment exerts numerous beneficial effects on functional, physical, and psychosocial outcomes, including muscle wasting and fatigue, metabolic and physical dysfunction, cognitive impairment, and depression [242,243,244,245]. To obtain health benefits, a combined protocol of intensive aerobic exercise of at least 75 min/week, or a moderate one of at least 150 min/week, with resistance training of major muscle groups 2–3 days per week for adults, has been recommended by the American College of Sports Medicine [246,247]. Alternatively, since cachectic patients may present physical limitations to performing exercise, several exercise mimetics are under investigation in preclinical trials to test their effectiveness to counteract dysmetabolism, overall preventing/delaying cachexia in cancer patients [248]. Among them, agonists of *P*PARδ or of AMPK, molecules able to activate SIRT1 or to reduce fatty acid oxidation, have given promising results to modulate lipid metabolism, mitochondrial function, and fiber-type determination in different muscle-wasting diseases, and cachexia as well [248,249]. Importantly, as part of a combined approach, exercise showed beneficial effects after chemotherapy in terms of reducing tumor growth rate, protein degradation and doxorubicin-induced toxicity in tumor-bearing mice [250].

## 5. Inter-or Transgenerational Effects of Nutrients and Exercise on Skeletal Muscle Mass

Parental nutritional support also influences future generations. Indeed, maternal low-protein diet [251,252] or high-fat diet (HFD) [253,254] consumption affect muscle metabolism and insulin sensitivity, predisposing the offspring to metabolic dysfunction later in life [255,256,257,258], in part by epigenetically altering skeletal muscle gene expression [259,260,261]. Moreover, maternal HFD correlates with compromised skeletal muscle development in the offspring, characterized by atrophy [253,262,263] and reduced performance [264,265], in part due to mitochondrial defects [263,266,267]. Even paternal nutrient support influences the metabolic state and the predisposition to metabolic disease [268,269], by epigenetic reprogramming of the sperm and offspring metabolic genes [270].

Importantly, maternal exercise can counteract the parental malnutrition-mediated effects in the offspring [271,272,273], by epigenetically modulating the expression of key regulators of skeletal muscle metabolism and mass, i.e., PGC-1α and the nuclear receptor Nr4a1 [271,274,275]. Interestingly, maternal exercise can even prevent the paternal obesity-induced metabolic dysfunction in the offspring skeletal muscle [276], in addition to paternal exercise [277].

Indeed, physical exercise is considered a physiological challenge whereby skeletal muscle has to remodel its metabolism, structure, and function in order to improve performance [278,279,280], via epigenetic regulation of gene transcription [281,282]. In addition, exercised muscles influence non-contractile tissues and whole-body metabolism by secreting myokines [283]. For these reasons, exercise is nowadays considered as a potential therapeutic approach for numerous diseases, including metabolic, oncological, and neurodegenerative diseases [13].

Recently, the beneficial effects of exercise have been shown to be transmitted to future generations, since parental physical exercise positively affects body metabolism, skeletal muscle structure, and performance of the offspring [263,273,284]. Studies from human and animal models demonstrated that maternal exercise performed during pregnancy increases muscle motility, reduces predisposition to obesity, and improves glucose homeostasis in the offspring [273,285,286,287,288]. Accumulating evidence indicates that paternal environmental exposure and physiology impact offspring development. In addition to increased sperm parameters, such as viability and motility [289,290], paternal exercise has been associated with increased mass and altered expression of metabolic genes in the offspring’s skeletal muscle [291,292].

Therefore, numerous studies have highlighted the important role of nutrients and parental physical exercise in the epigenetic regulation of offspring muscle metabolism. Additional studies are needed to clarify the long-term effects of parental exercise on offspring muscle metabolism and performance in humans. New advanced techniques, such as scanning X-ray micro-diffraction [293], can be exploited in the future to provide structural details on the skeletal muscle fibers.

## 6. Conclusions

In conclusion, available epidemiologic evidence supports the notion that many deleterious effects of physio-pathological conditions, such as sarcopenia, ALS, or cancer-induced cachexia, can be prevented by specific nutritional support or physical exercise. Moreover, acting on metabolic cues, through dietary interventions or physical exercise, may also be beneficial for the epigenetic regulation of muscle metabolism in the offspring. Therefore, nutritional support and exercise can be proposed as nonpharmacological approaches to ameliorate skeletal muscle homeostasis in numerous pathological states, by regulating protein synthesis, degradation, and metabolism. Despite encouraging data from animal studies supporting these alternative approaches, more clinical research is necessary to validate the potential of specific dietary treatments or exercise protocols.

## References

[1] Schiaffino S., Reggiani C. (2011). Fiber types in Mammalian skeletal muscles. Physiol. Rev..

[2] Pette D., Staron R.S. (2000). Myosin isoforms, muscle fiber types, and transitions. Microsc. Res. Tech..

[3] Estévez A., Andree K., Johnston I.A. (2012). Fast skeletal muscle transcriptome of the gilthead sea bream (*Sparus aurata*) determined by next generation sequencing. BMC Genom..

[4] Zhu J., Lu H., Xia B., Li Y., Li X., Zhang Q., Yang G. (2016). RNA-seq transcriptome analysis of extensor digitorum longus and soleus muscles in large white pigs. Mol. Genet. Genom..

[5] Ma J., Wang H., Liu R., Jin L., Tang Q., Wang X., Jiang A., Hu Y., Li Z., Zhu L. (2015). The miRNA Transcriptome Directly Reflects the Physiological and Biochemical Differences between Red, White, and Intermediate Muscle Fiber Types. Int. J. Mol. Sci..

[6] Johnson M., Polgar J., Weightman D., Appleton D. (1973). Data on the distribution of fibre types in thirty-six human muscles. An autopsy study. J. Neurol. Sci..

[7] Ciciliot S., Rossi A.C., Dyar K.A., Blaauw B., Schiaffino S. (2013). Muscle type and fiber type specificity in muscle wasting. Int. J. Biochem. Cell Biol..

[8] Wang Y., Pessin J.E. (2013). Mechanisms for fiber-type specificity of skeletal muscle atrophy. Curr. Opin. Clin. Nutr. Metab. Care.

[9] Stein T.P., Wade C.E. (2005). Metabolic consequences of muscle disuse atrophy. J. Nutr..

[10] Peggion C., Massimino M.L., Biancotto G., Angeletti R., Reggiani C., Sorgato M.C., Bertoli A., Stella R. (2017). Absolute quantification of myosin heavy chain isoforms by selected reaction monitoring can underscore skeletal muscle changes in a mouse model of amyotrophic lateral sclerosis. Anal. Bioanal. Chem..

[11] Scaricamazza S., Salvatori I., Giacovazzo G., Loeffler J.P., Renè F., Rosina M., Quessada C., Proietti D., Heil C., Rossi S. (2020). Skeletal-Muscle Metabolic Reprogramming in ALS-SOD1G93A Mice Predates Disease Onset and Is A Promising Therapeutic Target. iScience.

[12] Ucci S., Renzini A., Russi V., Mangialardo C., Cammarata I., Cavioli G., Santaguida M.G., Virili C., Centanni M., Adamo S. (2019). Thyroid hormone protects from fasting-induced skeletal muscle atrophy by promoting metabolic adaptation. Int. J. Mol. Sci..

[13] Pedersen B.K., Saltin B. (2015). Exercise as medicine—Evidence for prescribing exercise as therapy in 26 different chronic diseases. Scand. J. Med. Sci. Sports.

[14] Haba Y., Fujimura T., Oyama K., Kinoshita J., Miyashita T., Fushida S., Harada S., Ohta T. (2019). Effect of Oral Branched-Chain Amino Acids and Glutamine Supplementation on Skeletal Muscle Atrophy After Total Gastrectomy in Rat Model. J. Surg. Res..

[15] Moro T., Ebert S.M., Adams C.M., Rasmussen B.B. (2016). Amino Acid Sensing in Skeletal Muscle. Trends Endocrinol. Metab..

[16] Sánchez Riera C., Lozanoska-ochser B., Testa S., Fornetti E., Bouché M., Madaro L. (2021). Muscle diversity, heterogeneity, and gradients: Learning from sarcoglycanopathies. Int. J. Mol. Sci..

[17] Gomes M.J., Martinez P.F., Pagan L.U., Damatto R.L., Cezar M.D.M., Lima A.R.R., Okoshi K., Okoshi M.P. (2017). Skeletal muscle aging: Influence of oxidative stress and physical exercise. Oncotarget.

[18] Marzetti E., Leeuwenburgh C. (2006). Skeletal muscle apoptosis, sarcopenia and frailty at old age. Exp. Gerontol..

[19] Hiona A., Leeuwenburgh C. (2008). The role of mitochondrial DNA mutations in aging and sarcopenia: Implications for the mitochondrial vicious cycle theory of aging. Exp. Gerontol..

[20] Blatteis C.M. (2012). Age-dependent changes in temperature regulation—A mini review. Gerontology.

[21] Roubenoff R. (2003). Catabolism of aging: Is it an inflammatory process?. Curr. Opin. Clin. Nutr. Metab. Care.

[22] Liguori I., Russo G., Curcio F., Bulli G., Aran L., Della-Morte D., Gargiulo G., Testa G., Cacciatore F., Bonaduce D. (2018). Oxidative stress, aging, and diseases. Clin. Interv. Aging.

[23] Meng S.J., Yu L.J. (2010). Oxidative stress, molecular inflammation and sarcopenia. Int. J. Mol. Sci..

[24] Brinster R.L., Troike D.E. (1979). Requirements for blastocyst development in vitro. J. Anim. Sci..

[25] Jansen S., Pantaleon M., Kaye P.L. (2008). Characterization and regulation of monocarboxylate cotransporters Slc16a7 and Slc16a3 in preimplantation mouse embryos. Biol. Reprod..

[26] Martin K.L., Leese H.J. (1995). Role of glucose in mouse preimplantation embryo development. Mol. Reprod. Dev..

[27] Peterson C.M., Johannsen D.L., Ravussin E. (2012). Skeletal muscle mitochondria and aging: A review. J. Aging Res..

[28] Conley K.E., Amara C.E., Jubrias S.A., Marcinek D.J. (2007). Mitochondrial function, fibre types and ageing: New insights from human muscle in vivo. Exp. Physiol..

[29] Johnson M.L., Robinson M.M., Nair S.K. (2013). Skeletal muscle aging and the mitochondrion. Trends Endocrinol. Metab..

[30] Crupi A.N., Nunnelee J.S., Taylor D.J., Thomas A., Vit P., Riera C.E., Gottlieb R.A., Goodridge H.S. (2018). Oxidative muscles have better mitochondrial homeostasis than glycolytic muscles throughout life and maintain mitochondrial function during aging. Aging.

[31] Larsson L., Sjödin B., Karlsson J. (1978). Histochemical and biochemical changes in human skeletal muscle with age in sedentary males, age 22–65 years. Acta Physiol. Scand..

[32] Larsson L. (1978). Morphological and functional characteristics of the ageing skeletal muscle in man. A cross-sectional study. Acta Physiol. Scand. Suppl..

[33] Coggan A.R., Spina R.J., King D.S., Rogers M.A., Rogers M.A., Brown M., Nemeth P.M., Holloszy J.O. (1992). Histochemical and enzymatic comparison of the gastrocnemius muscle of young and elderly men and women. J. Gerontol..

[34] Klitgaard H., Mantoni M., Schiaffino S., Ausoni S., Gorza L., Laurent-Winter C., Schnohr P., Saltin B. (1990). Function, morphology and protein expression of ageing skeletal muscle: A cross-sectional study of elderly men with different training backgrounds. Acta Physiol. Scand..

[35] Lexell J., Taylor C.C., Sjöström M. (1988). What is the cause of the ageing atrophy? Total number, size and proportion of different fiber types studied in whole vastus lateralis muscle from 15- to 83-year-old men. J. Neurol. Sci..

[36] Frontera W.R., Hughes V.A., Fielding R.A., Fiatarone M.A., Evans W.J., Roubenoff R. (2000). Aging of skeletal muscle: A 12-yr longitudinal study. J. Appl. Physiol..

[37] Andersen J.L. (2003). Muscle fibre type adaptation in the elderly human muscle. Scand. J. Med. Sci. Sports.

[38] Campbell M.J., McComas A.J., Petito F. (1973). Physiological changes in ageing muscles. J. Neurol. Neurosurg. Psychiatry.

[39] Flück M., Hoppeler H. (2003). Molecular basis of skeletal muscle plasticity—from gene to form and function. Rev. Physiol. Biochem. Pharmacol..

[40] Seene T., Kaasik P., Riso E.M. (2012). Review on aging, unloading and reloading: Changes in skeletal muscle quantity and quality. Arch. Gerontol. Geriatr..

[41] Henze H., Jung M.J., Ahrens H.E., Steiner S., von Maltzahn J. (2020). Skeletal muscle aging—Stem cells in the spotlight. Mech. Ageing Dev..

[42] Carey B.W., Finley L.W.S., Cross J.R., Allis C.D., Thompson C.B. (2015). Intracellular α-ketoglutarate maintains the pluripotency of embryonic stem cells. Nature.

[43] Moussaieff A., Kogan N.M., Aberdam D. (2015). Concise Review: Energy Metabolites: Key Mediators of the Epigenetic State of Pluripotency. Stem Cells.

[44] Ryall J.G., Cliff T., Dalton S., Sartorelli V. (2015). Metabolic Reprogramming of Stem Cell Epigenetics. Cell Stem Cell.

[45] Harvey A., Caretti G., Moresi V., Renzini A., Adamo S. (2019). Interplay between Metabolites and the Epigenome in Regulating Embryonic and Adult Stem Cell Potency and Maintenance. Stem Cell Rep..

[46] Yamakawa H., Kusumoto D., Hashimoto H., Yuasa S. (2020). Stem cell aging in skeletal muscle regeneration and disease. Int. J. Mol. Sci..

[47] Bernet J.D., Doles J.D., Hall J.K., Kelly Tanaka K., Carter T.A., Olwin B.B. (2014). P38 MAPK signaling underlies a cell-autonomous loss of stem cell self-renewal in skeletal muscle of aged mice. Nat. Med..

[48] Solanas G., Peixoto F.O., Perdiguero E., Jardí M., Ruiz-Bonilla V., Datta D., Symeonidi A., Castellanos A., Welz P.-S., Caballero J.M. (2017). Aged Stem Cells Reprogram Their Daily Rhythmic Functions to Adapt to Stress. Cell.

[49] Nieuwenhuizen W.F., Weenen H., Rigby P., Hetherington M.M. (2010). Older adults and patients in need of nutritional support: Review of current treatment options and factors influencing nutritional intake. Clin. Nutr..

[50] Robinson S., Cooper C., Aihie Sayer A. (2012). Nutrition and sarcopenia: A review of the evidence and implications for preventive strategies. J. Aging Res..

[51] Sarkar T.J., Quarta M., Mukherjee S., Colville A., Paine P., Doan L., Tran C.M., Chu C.R., Horvath S., Qi L.S. (2020). Transient non-integrative expression of nuclear reprogramming factors promotes multifaceted amelioration of aging in human cells. Nat. Commun..

[52] Kaiser M.J., Bandinelli S., Lunenfeld B. (2010). Frailty and the role of nutrition in older people: A review of the current literature. Acta Biomed..

[53] Kupisz-Urbańska M., Płudowski P., Marcinowska-Suchowierska E. (2021). Vitamin D deficiency in older patients—Problems of sarcopenia, drug interactions, management in deficiency. Nutrients.

[54] Abiri B., Vafa M. (2020). Vitamin D and Muscle Sarcopenia in Aging. Methods Mol. Biol..

[55] Rondanelli M., Klersy C., Terracol G., Talluri J., Maugeri R., Guido D., Faliva M.A., Solerte B.S., Fioravanti M., Lukaski H. (2016). Whey protein, amino acids, and Vitamin D supplementation with physical activity increases fat-free mass and strength, functionality, and quality of life and decreases inflammation in sarcopenic elderly. Am. J. Clin. Nutr..

[56] Verlaan S., Maier A.B., Bauer J.M., Bautmans I., Brandt K., Donini L.M., Maggio M., McMurdo M.E.T., Mets T., Seal C. (2018). Sufficient levels of 25-hydroxyvitamin D and protein intake required to increase muscle mass in sarcopenic older adults—The PROVIDE study. Clin. Nutr..

[57] Kim J.S., Wilson J.M., Lee S.R. (2010). Dietary implications on mechanisms of sarcopenia: Roles of protein, amino acids and antioxidants. J. Nutr. Biochem..

[58] Drummond M.J., Dickinson J.M., Fry C.S., Walker D.K., Gundermann D.M., Reidy P.T., Timmerman K.L., Markofski M.M., Paddon-Jones D., Rasmussen B.B. (2012). Bed rest impairs skeletal muscle amino acid transporter expression, mTORC1 signaling, and protein synthesis in response to essential amino acids in older adults. Am. J. Physiol. Endocrinol. Metab..

[59] Bidon C., Lachuer J., Molgó J., Wierinckx A., de la Porte S., Pignol B., Christen Y., Meloni R., Koenig H., Biguet N.F. (2009). The extract of Ginkgo biloba EGb 761 reactivates a juvenile profile in the skeletal muscle of sarcopenic rats by transcriptional reprogramming. PLoS ONE.

[60] Lv Y., Liang T., Wang G., Li Z. (2018). Ghrelin, A gastrointestinal hormone, regulates energy balance and lipid metabolism. Biosci. Rep..

[61] Seyhanli E.S., Lok U., Gulacti U., Buyukaslan H., Atescelik M., Yildiz M., Onur M.R., Goktekin M.C., Aydın S. (2015). Assessment of serum and urine ghrelin levels in patients with acute stroke. Int. J. Clin. Exp. Med..

[62] Porporato P.E., Filigheddu N., Reano S., Ferrara M., Angelino E., Gnocchi V.F., Prodam F., Ronchi G., Fagoonee S., Fornaro M. (2013). Acylated and unacylated ghrelin impair skeletal muscle atrophy in mice. J. Clin. Investig..

[63] Amitani M., Amitani H., Cheng K.C., Kairupan T.S., Sameshima N., Shimoshikiryo I., Mizuma K., Rokot N.T., Nerome Y., Owaki T. (2017). The role of ghrelin and ghrelin signaling in aging. Int. J. Mol. Sci..

[64] Brook M.S., Wilkinson D.J., Phillips B.E., Perez-Schindler J., Philp A., Smith K., Atherton P.J. (2016). Skeletal muscle homeostasis and plasticity in youth and ageing: Impact of nutrition and exercise. Acta Physiol..

[65] Bowen T.S., Schuler G., Adams V. (2015). Skeletal muscle wasting in cachexia and sarcopenia: Molecular pathophysiology and impact of exercise training. J. Cachexia Sarcopenia Muscle.

[66] Hornberger T.A., Chu W.K., Mak Y.W., Hsiung J.W., Huang S.A., Chien S. (2006). The role of phospholipase D and phosphatidic acid in the mechanical activation of mTOR signaling in skeletal muscle. Proc. Natl. Acad. Sci. USA.

[67] O’neil T.K., Duffy L.R., Frey J.W., Hornberger T.A. (2009). The role of phosphoinositide 3-kinase and phosphatidic acid in the regulation of mammalian target of rapamycin following eccentric contractions. J. Physiol..

[68] Klossner S., Durieux A.C., Freyssenet D., Flueck M. (2009). Mechano-transduction to muscle protein synthesis is modulated by FAK. Eur. J. Appl. Physiol..

[69] Jones A.M., Carter H. (2000). The effect of endurance training on parameters of aerobic fitness. Sports Med..

[70] Di Donato D.M., West D.W.D., Churchward-Venne T.A., Breen L., Baker S.K., Phillips S.M. (2014). Influence of aerobic exercise intensity on myofibrillar and mitochondrial protein synthesis in young men during early and late postexercise recovery. Am. J. Physiol. Endocrinol. Metab..

[71] Liang J., Zhang H., Zeng Z., Wu L., Zhang Y., Guo Y., Lv J., Wang C., Fan J., Chen N. (2021). Lifelong Aerobic Exercise Alleviates Sarcopenia by Activating Autophagy and Inhibiting Protein Degradation via the AMPK/PGC-1α Signaling Pathway. Metabolites.

[72] Rooyackers O.E., Adey D.B., Ades P.A., Nair K.S. (1996). Effect of age on in vivo rates of mitochondrial protein synthesis in human skeletal muscle. Proc. Natl. Acad. Sci. USA.

[73] Muddapu V.R., Dharshini S.A.P., Chakravarthy V.S., Gromiha M.M. (2020). Neurodegenerative Diseases—Is Metabolic Deficiency the Root Cause?. Front. Neurosci..

[74] Hardiman O., Al-Chalabi A., Chio A., Corr E.M., Logroscino G., Robberecht W., Shaw P.J., Simmons Z., Van Den Berg L.H. (2017). Amyotrophic lateral sclerosis. Nat. Rev. Dis. Prim..

[75] Dupuis L., Pradat P.F., Ludolph A.C., Loeffler J.P. (2011). Energy metabolism in amyotrophic lateral sclerosis. Lancet Neurol..

[76] Joardar A., Manzo E., Zarnescu D.C. (2017). Metabolic Dysregulation in Amyotrophic Lateral Sclerosis: Challenges and Opportunities. Curr. Genet. Med. Rep..

[77] Steyn F.J., Li R., Kirk S.E., Tefera T.W., Xie T.Y., Tracey T.J., Kelk D., Wimberger E., Garton F.C., Roberts L. (2020). Altered skeletal muscle glucose-fatty acid flux in amyotrophic lateral sclerosis. Brain Commun..

[78] Palamiuc L., Schlagowski A., Ngo S.T., Vernay A., Dirrig-Grosch S., Henriques A., Boutillier A., Zoll J., Echaniz-Laguna A., Loeffler J. (2015). A metabolic switch toward lipid use in glycolytic muscle is an early pathologic event in a mouse model of amyotrophic lateral sclerosis. EMBO Mol. Med..

[79] Desseille C., Deforges S., Biondi O., Houdebine L., D’Amico D., Lamazière A., Caradeuc C., Bertho G., Bruneteau G., Weill L. (2017). Specific physical exercise improves energetic metabolism in the skeletal muscle of amyotrophic-lateral- sclerosis mice. Front. Mol. Neurosci..

[80] Smittkamp S.E., Morris J.K., Bomhoff G.L., Chertoff M.E., Geiger P.C., Stanford J.A. (2013). SOD1-G93A mice exhibit muscle-fiber-type-specific decreases in glucose uptake in the absence of whole-body changes in metabolism. Neurodegener. Dis..

[81] White M.F. (2002). IRS proteins and the common path to diabetes. Am. J. Physiol. Endocrinol. Metab..

[82] Dobrowolny G., Aucello M., Rizzuto E., Beccafico S., Mammucari C., Boncompagni S., Belia S., Wannenes F., Nicoletti C., Del Prete Z. (2008). Skeletal Muscle Is a Primary Target of SOD1G93A-Mediated Toxicity. Cell Metab..

[83] Dobrowolny G., Lepore E., Martini M., Barberi L., Nunn A., Scicchitano B.M., Musarò A. (2018). Metabolic changes associated with muscle expression of SOD1G93A. Front. Physiol..

[84] Begani Provinciali G., Pieroni N., Bukreeva I., Fratini M., Massimi L., Maugeri L., Palermo F., Bardelli F., Mittone A., Bravin A. (2020). X-ray phase contrast tomography for the investigation of amyotrophic lateral sclerosis. J. Synchrotron Radiat..

[85] Tefera T.W., Steyn F.J., Ngo S.T., Borges K. (2021). CNS glucose metabolism in Amyotrophic Lateral Sclerosis: A therapeutic target?. Cell Biosci..

[86] Park Y., Park J., Kim Y., Baek H., Kim S.H. (2015). Association between nutritional status and disease severity using the amyotrophic lateral sclerosis (ALS) functional rating scale in ALS patients. Nutrition.

[87] Paganoni S., Deng J., Jaffa M., Cudkowicz M.E., Wills A.M. (2011). Body mass index, not dyslipidemia, is an independent predictor of survival in amyotrophic lateral sclerosis. Muscle Nerve.

[88] Reich-Slotky R., Andrews J., Cheng B., Buchsbaum R., Levy D., Kaufmann P., Thompson J.L.P. (2013). Body mass index (BMI) as predictor of ALSFRS-R score decline in ALS patients. Amyotroph. Lateral Scler. Front. Degener..

[89] Longinetti E., Mariosa D., Larsson H., Ye W., Ingre C., Almqvist C., Lichtenstein P., Piehl F., Fang F. (2017). Neurodegenerative and psychiatric diseases among families with amyotrophic lateral sclerosis. Neurology.

[90] Goodpaster B.H., Sparks L.M. (2017). Metabolic Flexibility in Health and Disease. Cell Metab..

[91] Fergani A., Oudart H., De Aguilar J.L.G., Fricker B., René F., Hocquette J.F., Meininger V., Dupuis L., Loeffler J.P. (2007). Increased peripheral lipid clearance in an animal model of amyotrophic lateral sclerosis. J. Lipid Res..

[92] Turner N., Cooney G.J., Kraegen E.W., Bruce C.R. (2014). Fatty acid metabolism, energy expenditure and insulin resistance in muscle. J. Endocrinol..

[93] Steyn F.J., Ioannides Z.A., Van Eijk R.P.A., Heggie S., Thorpe K.A., Ceslis A., Heshmat S., Henders A.K., Wray N.R., Van Den Berg L.H. (2018). Hypermetabolism in ALS is associated with greater functional decline and shorter survival. J. Neurol. Neurosurg. Psychiatry.

[94] Wills A.M., Hubbard J., Macklin E.A., Glass J., Tandan R., Simpson E.P., Brooks B., Gelinas D., Mitsumoto H., Mozaffar T. (2014). Hypercaloric enteral nutrition in patients with amyotrophic lateral sclerosis: A randomised, double-blind, placebo-controlled phase 2 trial. Lancet.

[95] Veldink J.H., Kalmijn S., Groeneveld G.J., Wunderink W., Koster A., De Vries J.H.M., Van Der Luyt J., Wokke J.H.J., Van Den Berg L.H. (2007). Intake of polyunsaturated fatty acids and vitamin E reduces the risk of developing amyotrophic lateral sclerosis. J. Neurol. Neurosurg. Psychiatry.

[96] Fitzgerald K.C., O’Reilly É.J., Falcone G.J., McCullough M.L., Park Y., Kolonel L.N., Ascherio A. (2014). Dietary ω-3 polyunsaturated fatty acid intake and risk for amyotrophic lateral sclerosis. JAMA Neurol..

[97] Ludolph A.C., Dorst J., Dreyhaupt J., Weishaupt J.H., Kassubek J., Weiland U., Meyer T., Petri S., Hermann A., Emmer A. (2020). Effect of High-Caloric Nutrition on Survival in Amyotrophic Lateral Sclerosis. Ann. Neurol..

[98] Zhao Z., Sui Y., Gao W., Cai B., Fan D. (2015). Effects of diet on adenosine monophosphate-activated protein kinase activity and disease progression in an amyotrophic lateral sclerosis model. J. Int. Med. Res..

[99] Coughlan K.S., Halang L., Woods I., Prehn J.H.M. (2016). A high-fat jelly diet restores bioenergetic balance and extends lifespan in the presence of motor dysfunction and lumbar spinal cord motor neuron loss in TDP-43A315T mutant C57BL6/J mice. DMM Dis. Model. Mech..

[100] Pedersen W.A., Mattson M.P. (1999). No benefit of dietary restriction on disease onset or progression in amyotrophic lateral sclerosis Cu/Zn-superoxide dismutase mutant mice. Brain Res..

[101] Gracies J.M. (2005). Pathophysiology of spastic paresis. II: Emergence of muscle overactivity. Muscle Nerve.

[102] Wang P., Deng J., Dong J., Liu J., Bigio E.H., Mesulam M., Wang T., Sun L., Wang L., Lee A.Y.L. (2019). TDP-43 induces mitochondrial damage and activates the mitochondrial unfolded protein response. PLoS Genet..

[103] Browne S.E., Bowling A.C., Baik M.J., Gurney M., Brown R.H., Beal M.F. (1998). Metabolic dysfunction in familial, but not sporadic, amyotrophic lateral sclerosis. J. Neurochem..

[104] Zhao Z., Lange D.J., Voustianiouk A., MacGrogan D., Ho L., Suh J., Humala N., Thiyagarajan M., Wang J., Pasinetti G.M. (2006). A ketogenic diet as a potential novel therapeutic intervention in amyotrophic lateral sclerosis. BMC Neurosci..

[105] Zhao W., Varghese M., Vempati P., Dzhun A., Cheng A., Wang J., Lange D., Bilski A., Faravelli I., Pasinetti G.M. (2012). Caprylic Triglyceride as a Novel Therapeutic Approach to Effectively Improve the Performance and Attenuate the Symptoms Due to the Motor Neuron Loss in ALS Disease. PLoS ONE.

[106] Romijn J.A., Coyle E.F., Sidossis L.S., Gastaldelli A., Horowitz J.F., Endert E., Wolfe R.R. (1993). Regulation of endogenous fat and carbohydrate metabolism in relation to exercise intensity and duration. Am. J. Physiol..

[107] Van Loon L.J., Greenhaff P.L., Constantin-Teodosiu D., Saris W.H., Wagenmakers A.J. (2001). The effects of increasing exercise intensity on muscle fuel utilisation in humans. J. Physiol..

[108] Memme J.M., Erlich A.T., Phukan G., Hood D.A. (2021). Exercise and mitochondrial health. J. Physiol..

[109] Kurtzke J.F. (1991). Risk factors in amyotrophic lateral sclerosis. Adv. Neurol..

[110] Strickland D., Smith S.A., Dolliff G., Goldman L., Roelofs R.I. (1996). Amyotrophic lateral sclerosis and occupational history: A pilot case- control study. Arch. Neurol..

[111] Scarmeas N., Shih T., Stern Y., Ottman R., Rowland L.P. (2002). Premorbid weight, body mass, and varsity athletics in ALS. Neurology.

[112] Julian T.H., Glascow N., Barry A.D.F., Moll T., Harvey C., Klimentidis Y.C., Newell M., Zhang S., Snyder M.P., Cooper-Knock J. (2021). Physical exercise is a risk factor for amyotrophic lateral sclerosis: Convergent evidence from Mendelian randomisation, transcriptomics and risk genotypes. EBioMedicine.

[113] Veldink J.H., Kalmijn S., Van Der Hout A.H., Lemmink H.H., Groeneveld G.J., Lummen C., Scheffer H., Wokke J.H.J., Van Den Berg L.H. (2005). SMN genotypes producing less SMN protein increase susceptibility to and severity of sporadic ALS. Neurology.

[114] Arnon R., Aharoni R. (2007). Neurogenesis and neuroprotection in the CNS—Fundamental elements in the effect of glatiramer acetate on treatment of autoimmune neurological disorders. Mol. Neurobiol..

[115] Huisman M.H., Seelen M., de Jong S.W., Dorresteijn K.R.I.S., van Doormaal P.T.C., van der Kooi A.J., de Visser M., Jurgen Schelhaas H., van den Berg L.H., Herman Veldink J. (2013). Lifetime physical activity and the risk of amyotrophic lateral sclerosis. J. Neurol. Neurosurg. Psychiatry.

[116] Pinto A.C., Alves M., Nogueira A., Evangelista T., Carvalho J., Coelho A., De Carvalho M., Sales-Luís M.L. (1999). Can amyotrophic lateral sclerosis patients with respiratory insufficiency exercise?. J. Neurol. Sci..

[117] Drory V.E., Goltsman E., Goldman Reznik J., Mosek A., Korczyn A.D. (2001). The value of muscle exercise in patients with amyotrophic lateral sclerosis. J. Neurol. Sci..

[118] Bello-Haas V.D., Florence J.M., Kloos A.D., Scheirbecker J., Lopate G., Hayes S.M., Pioro E.P., Mitsumoto H. (2007). A randomized controlled trial of resistance exercise in individuals with ALS. Neurology.

[119] Kirkinezos I.G., Hernandez D., Bradley W.G., Moraes C.T. (2003). Regular exercise is beneficial to a mouse model of amyotrophic lateral sclerosis. Ann. Neurol..

[120] Gerber Y.N., Sabourin J.C., Hugnot J.P., Perrin F.E. (2012). Unlike Physical Exercise, Modified Environment Increases the Lifespan of SOD1G93A Mice However Both Conditions Induce Cellular Changes. PLoS ONE.

[121] Ortega-Hombrados L., Molina-Torres G., Galán-Mercant A., Sánchez-Guerrero E., González-Sánchez M., Ruiz-Muñoz M. (2021). Systematic Review of Therapeutic Physical Exercise in Patients with Amyotrophic Lateral Sclerosis over Time. Int. J. Environ. Res. Public Health.

[122] Fearon K., Strasser F., Anker S.D., Bosaeus I., Bruera E., Fainsinger R.L., Jatoi A., Loprinzi C., MacDonald N., Mantovani G. (2011). Definition and classification of cancer cachexia: An international consensus. Lancet Oncol..

[123] Porporato P.E. (2016). Understanding cachexia as a cancer metabolism syndrome. Oncogenesis.

[124] Sartori R., Romanello V., Sandri M. (2021). Mechanisms of muscle atrophy and hypertrophy: Implications in health and disease. Nat. Commun..

[125] Bonaldo P., Sandri M. (2013). Cellular and molecular mechanisms of muscle atrophy. DMM Dis. Model. Mech..

[126] Cohen S., Nathan J.A., Goldberg A.L. (2014). Muscle wasting in disease: Molecular mechanisms and promising therapies. Nat. Rev. Drug Discov..

[127] Costelli P., Muscaritoli M., Bossola M., Penna F., Reffo P., Bonetto A., Busquets S., Bonelli G., Lopez-Soriano F.J., Doglietto G.B. (2006). IGF-1 is downregulated in experimental cancer cachexia. Am. J. Physiol. Regul. Integr. Comp. Physiol..

[128] Asp M.L., Tian M., Wendel A.A., Belury M.A. (2010). Evidence for the contribution of insulin resistance to the development of cachexia in tumor-bearing mice. Int. J. Cancer.

[129] Rofe A.M., Bourgeois C.S., Coyle P., Taylor A., Abdi E.A. (1994). Altered insulin response to glucose in weight-losing cancer patients. Anticancer Res..

[130] Mantovani G., Macciò A., Mura L., Massa E., Mudu M.C., Mulas C., Lusso M.R., Madeddu C., Dessì A. (2000). Serum levels of leptin and proinflammatory cytokines in patients with advanced-stage cancer at different sites. J. Mol. Med..

[131] He W.A., Calore F., Londhe P., Canella A., Guttridge D.C., Croce C.M. (2014). Microvesicles containing miRNAs promote muscle cell death in cancer cachexia via TLR7. Proc. Natl. Acad. Sci. USA.

[132] Qiao F., Pan P., Yan J., Sun J., Zong Y., Wu Z., Lu X., Chen N., Mi R., Ma Y. (2019). Role of tumor-derived extracellular vesicles in cancer progression and their clinical applications (Review). Int. J. Oncol..

[133] Daou N., Hassani M., Matos E., De Castro G.S., Costa R.G.F., Seelaender M., Moresi V., Rocchi M., Adamo S., Li Z. (2020). Displaced myonuclei in cancer cachexia suggest altered innervation. Int. J. Mol. Sci..

[134] Boehm I., Miller J., Wishart T.M., Wigmore S.J., Skipworth R.J.E., Jones R.A., Gillingwater T.H. (2020). Neuromuscular junctions are stable in patients with cancer cachexia. J. Clin. Investig..

[135] Fermoselle C., García-Arumí E., Puig-Vilanova E., Andreu A.L., Urtreger A.J., de Kier Joffé E.D.B., Tejedor A., Puente-Maestu L., Barreiro E. (2013). Mitochondrial dysfunction and therapeutic approaches in respiratory and limb muscles of cancer cachectic mice. Exp. Physiol..

[136] Aria Tzika A., Fontes-Oliveira C.C., Shestov A.A., Constantinou C., Psychogios N., Righi V., Mintzopoulos D., Busquets S., Lopez-Soriano F.J., Milot S. (2013). Skeletal muscle mitochondrial uncoupling in a murine cancer cachexia model. Int. J. Oncol..

[137] Antunes D., Padrão A.I., Maciel E., Santinha D., Oliveira P., Vitorino R., Moreira-Gonçalves D., Colaço B., Pires M.J., Nunes C. (2014). Molecular insights into mitochondrial dysfunction in cancer-related muscle wasting. Biochim. Biophys. Acta Mol. Cell Biol. Lipids.

[138] Neyroud D., Nosacka R.L., Judge A.R., Hepple R.T. (2019). Colon 26 adenocarcinoma (C26)-induced cancer cachexia impairs skeletal muscle mitochondrial function and content. J. Muscle Res. Cell Motil..

[139] Barreiro E., De La Puente B., Busquets S., López-Soriano F.J., Gea J., Argilés J.M. (2005). Both oxidative and nitrosative stress are associated with muscle wasting in tumour-bearing rats. FEBS Lett..

[140] Sullivan-Gunn M.J., Campbell-O’Sullivan S.P., Tisdale M.J., Lewandowski P.A. (2011). Decreased NADPH oxidase expression and antioxidant activity in cachectic skeletal muscle. J. Cachexia Sarcopenia Muscle.

[141] McLean J.B., Moylan J.S., Andrade F.H. (2014). Mitochondria dysfunction in lung cancer-induced muscle wasting in C2C12 myotubes. Front. Physiol..

[142] Cancer Research Differential Reconstitution of Mitochondrial Respiratory Chain Activity and Plasma Redox State by Cysteine and Ornithine in a Model of Cancer Cachexia. https://cancerres.aacrjournals.org/content/59/14/3527.long.

[143] Brown J.L., Rosa-Caldwell M.E., Lee D.E., Blackwell T.A., Brown L.A., Perry R.A., Haynie W.S., Hardee J.P., Carson J.A., Wiggs M.P. (2017). Mitochondrial degeneration precedes the development of muscle atrophy in progression of cancer cachexia in tumour-bearing mice. J. Cachexia Sarcopenia Muscle.

[144] Vanderveen B.N., Fix D.K., Carson J.A. (2017). Disrupted Skeletal Muscle Mitochondrial Dynamics, Mitophagy, and Biogenesis during Cancer Cachexia: A Role for Inflammation. Oxid. Med. Cell. Longev..

[145] Argilés J.M., Fontes-Oliveira C.C., Toledo M., López-Soriano F.J., Busquets S. (2014). Cachexia: A problem of energetic inefficiency. J. Cachexia Sarcopenia Muscle.

[146] Sanchís D., Busquets S., Alvarez B., Ricquier D., López-Soriano F.J., Argilés J.M. (1998). Skeletal muscle UCP2 and UCP3 gene expression in a rat cancer cachexia model. FEBS Lett..

[147] Montgomery M.K., Turner N. (2015). Mitochondrial dysfunction and insulin resistance: An update. Endocr. Connect..

[148] Unger R.H. (2003). Minireview: Weapons of Lean Body Mass Destruction: The Role of Ectopic Lipids in the Metabolic Syndrome. Endocrinology.

[149] Koopman R., Van Loon L.J.C. (2009). Aging, exercise, and muscle protein metabolism. J. Appl. Physiol..

[150] Johns N., Hatakeyama S., Stephens N.A., Degen M., Degen S., Frieauff W., Lambert C., Ross J.A., Roubenoff R., Glass D.J. (2014). Clinical classification of cancer cachexia: Phenotypic correlates in human skeletal muscle. PLoS ONE.

[151] Taskin S., Stumpf V.I., Bachmann J., Weber C., Martignoni M.E., Friedrich O. (2014). Motor protein function in skeletal abdominal muscle of cachectic cancer patients. J. Cell. Mol. Med..

[152] Op den Kamp C.M., Gosker H.R., Lagarde S., Tan D.Y., Snepvangers F.J., Dingemans A.M.C., Langen R.C.J., Schols A.M.W.J. (2015). Preserved muscle oxidative metabolic phenotype in newly diagnosed non-small cell lung cancer cachexia. J. Cachexia Sarcopenia Muscle.

[153] Aulino P., Berardi E., Cardillo V.M., Rizzuto E., Perniconi B., Ramina C., Padula F., Spugnini E.P., Baldi A., Faiola F. (2010). Molecular, cellular and physiological characterization of the cancer cachexia-inducing C26 colon carcinoma in mouse. BMC Cancer.

[154] Stephens N.A., Skipworth R.J.E., MacDonald A.J., Greig C.A., Ross J.A., Fearon K.C.H. (2011). Intramyocellular lipid droplets increase with progression of cachexia in cancer patients. J. Cachexia Sarcopenia Muscle.

[155] Almasud A.A., Giles K.H., Miklavcic J.J., Martins K.J.B., Baracos V.E., Putman C.T., Guan L.L., Mazurak V.C. (2017). Fish oil mitigates myosteatosis and improves chemotherapy efficacy in a preclinical model of colon cancer. PLoS ONE.

[156] van Dijk D.P.J., Bakens M.J.A.M., Coolsen M.M.E., Rensen S.S., van Dam R.M., Bours M.J.L., Weijenberg M.P., Dejong C.H.C., Olde Damink S.W.M. (2017). Low skeletal muscle radiation attenuation and visceral adiposity are associated with overall survival and surgical site infections in patients with pancreatic cancer. J. Cachexia Sarcopenia Muscle.

[157] Narasimhan A., Ghosh S., Stretch C., Greiner R., Bathe O.F., Baracos V., Damaraju S. (2017). Small RNAome profiling from human skeletal muscle: Novel miRNAs and their targets associated with cancer cachexia. J. Cachexia Sarcopenia Muscle.

[158] Johns N., Stretch C., Tan B.H.L., Solheim T.S., Sørhaug S., Stephens N.A., Gioulbasanis I., Skipworth R.J.E., Deans D.A.C., Vigano A. (2017). New genetic signatures associated with cancer cachexia as defined by low skeletal muscle index and weight loss. J. Cachexia Sarcopenia Muscle.

[159] Narasimhan A., Greiner R., Bathe O.F., Baracos V., Damaraju S. (2018). Differentially expressed alternatively spliced genes in skeletal muscle from cancer patients with cachexia. J. Cachexia Sarcopenia Muscle.

[160] Von Haehling S., Anker S.D. (2010). Cachexia as a major underestimated and unmet medical need: Facts and numbers. J. Cachexia Sarcopenia Muscle.

[161] Zhou X., Wang J.L., Lu J., Song Y., Kwak K.S., Jiao Q., Rosenfeld R., Chen Q., Boone T., Simonet W.S. (2010). Reversal of cancer cachexia and muscle wasting by ActRIIB antagonism leads to prolonged survival. Cell.

[162] Pigna E., Berardi E., Aulino P., Rizzuto E., Zampieri S., Carraro U., Kern H., Merigliano S., Gruppo M., Mericskay M. (2016). Aerobic Exercise and Pharmacological Treatments Counteract Cachexia by Modulating Autophagy in Colon Cancer. Sci. Rep..

[163] Solheim T.S., Laird B.J.A. (2012). Evidence base for multimodal therapy in cachexia. Curr. Opin. Support. Palliat. Care.

[164] Baldwin C., Weekes C.E. (2011). Dietary advice with or without oral nutritional supplements for disease-related malnutrition in adults. Cochrane Database Syst. Rev..

[165] Wallengren O., Lundholm K., Bosaeus I. (2005). Diet energy density and energy intake in palliative care cancer patients. Clin. Nutr..

[166] Engelen M.P.K.J., Safar A.M., Bartter T., Koeman F., Deutz N.E.P. (2015). High anabolic potential of essential amino acid mixtures in advanced nonsmall cell lung cancer. Ann. Oncol. Off. J. Eur. Soc. Med. Oncol..

[167] Fujita S., Dreyer H.C., Drummond M.J., Glynn E.L., Cadenas J.G., Yoshizawa F., Volpi E., Rasmussen B.B. (2007). Nutrient signalling in the regulation of human muscle protein synthesis. J. Physiol..

[168] Den Kamp C.M.O., Langen R.C., Haegens A., Schols A.M. (2009). Muscle atrophy in cachexia: Can dietary protein tip the balance?. Curr. Opin. Clin. Nutr. Metab. Care.

[169] Arends J., Bachmann P., Baracos V., Barthelemy N., Bertz H., Bozzetti F., Fearon K., Hütterer E., Isenring E., Kaasa S. (2017). ESPEN guidelines on nutrition in cancer patients. Clin. Nutr..

[170] Huang Y., Cao D., Chen Z., Chen B., Li J., Guo J., Dong Q., Liu L., Wei Q. (2021). Red and processed meat consumption and cancer outcomes: Umbrella review. Food Chem..

[171] Klement R.J., Brehm N., Sweeney R.A. (2020). Ketogenic diets in medical oncology: A systematic review with focus on clinical outcomes. Med. Oncol..

[172] Ruiz-García V., López-Briz E., Carbonell-Sanchis R., Bort-Martí S., Gonzálvez-Perales J.L. (2018). Megestrol acetate for cachexia–anorexia syndrome. A systematic review. J. Cachexia Sarcopenia Muscle.

[173] Esfahani M., Sahafi S., Derakhshandeh A., Moghaddas A. (2018). The anti-wasting effects of L-carnitine supplementation on cancer: Experimental data and clinical studies. Asia Pac. J. Clin. Nutr..

[174] Bar-Sela G., Zalman D., Semenysty V., Ballan E. (2019). The Effects of Dosage-Controlled Cannabis Capsules on Cancer-Related Cachexia and Anorexia Syndrome in Advanced Cancer Patients: Pilot Study. Integr. Cancer Ther..

[175] Khatib M.N., Gaidhane A., Gaidhane S., Quazi Z.S. (2018). Ghrelin as a promising therapeutic option for cancer cachexia. Cell. Physiol. Biochem..

[176] Siff T., Parajuli P., Razzaque M.S., Atfi A. (2021). Cancer-Mediated Muscle Cachexia: Etiology and Clinical Management. Trends Endocrinol. Metab..

[177] Mori T., Ohmori H., Luo Y., Mori S., Miyagawa Y., Nukaga S., Goto K., Fujiwara-Tani R., Kishi S., Sasaki T. (2019). Giving combined medium-chain fatty acids and glucose protects against cancer-associated skeletal muscle atrophy. Cancer Sci..

[178] Gorjao R., dos Santos C.M.M., Serdan T.D.A., Diniz V.L.S., Alba-Loureiro T.C., Cury-Boaventura M.F., Hatanaka E., Levada-Pires A.C., Sato F.T., Pithon-Curi T.C. (2019). New insights on the regulation of cancer cachexia by N-3 polyunsaturated fatty acids. Pharmacol. Ther..

[179] Yan Z., Zhong Y., Duan Y., Chen Q., Li F. (2020). Antioxidant mechanism of tea polyphenols and its impact on health benefits. Anim. Nutr..

[180] Penna F., Camperi A., Muscaritoli M., Filigheddu N., Costelli P. (2017). The role of Vitamin D in cancer cachexia. Curr. Opin. Support. Palliat. Care.

[181] Tisdale M.J. (1993). Mechanism of lipid mobilization associated with cancer cachexia: Interaction between the polyunsaturated fatty acid, eicosapentaenoic acid, and inhibitory guanine nucleotide-regulatory protein. Prostaglandins Leukot. Essent. Fat. Acids.

[182] Anti M., Marra G., Armelao F., Bartoli G.M., Ficarelli R., Percesepe A., De Vitis I., Maria G., Sofo L., Rapaccini G.L. (1992). Effect of ω-3 fatty acids on rectal mucosal cell proliferation in subjects at risk for colon cancer. Gastroenterology.

[183] Rose D.P., Connolly J.M. (1993). Effects of dietary omega-3 fatty acids on human breast cancer growth and metastases in nude mice. J. Natl. Cancer Inst..

[184] La Guardia M., Giammanco S., Di Majo D., Tabacchi G., Tripoli E., Giammanco M. (2005). Omega 3 Fatty Acids: Biological Activity and Effects on Human Health. Panminerva Med..

[185] Dunbar B.S., Bosire R.V., Deckelbaum R.J. (2014). Omega 3 and omega 6 fatty acids in human and animal health: An African perspective. Mol. Cell. Endocrinol..

[186] Barber M.D., Ross J.A., Voss A.C., Tisdale M.J., Fearon K.C.H. (1999). The effect of an oral nutritional supplement enriched with fish oil on weight-loss in patients with pancreatic cancer. Br. J. Cancer.

[187] Read J.A., Beale P.J., Volker D.H., Smith N., Childs A., Clarke S.J. (2007). Nutrition intervention using an eicosapentaenoic acid (EPA)-containing supplement in patients with advanced colorectal cancer. Effects on nutritional and inflammatory status: A phase II trial. Support. Care Cancer.

[188] Song M., Zhang X., Meyerhardt J.A., Giovannucci E.L., Ogino S., Fuchs C.S., Chan A.T. (2017). Marine ω-3 polyunsaturated fatty acid intake and survival after colorectal cancer diagnosis. Gut.

[189] Gogos C.A., Ginopoulos P., Salsa B., Apostolidou E., Zoumbos N.C., Kalfarentzos F. (1998). Dietary omega-3 polyunsaturated fatty acids plus vitamin E restore immunodeficiency and prolong survival for severely ill patients with generalized malignancy: A randomized control trial. Cancer.

[190] Lipina C., Hundal H.S. (2017). Lipid modulation of skeletal muscle mass and function. J. Cachexia Sarcopenia Muscle.

[191] Pinel A., Rigaudière J.P., Laillet B., Pouyet C., Malpuech-Brugère C., Prip-Buus C., Morio B., Capel F. (2016). N—3PUFA differentially modulate palmitate-induced lipotoxicity through alterations of its metabolism in C2C12 muscle cells. Biochim. Biophys. Acta-Mol. Cell Biol. Lipids.

[192] Smith G.I., Julliand S., Reeds D.N., Sinacore D.R., Klein S., Mittendorfer B. (2015). Fish oil-derived n-3 PUFA therapy increases muscle mass and function in healthy older adults. Am. J. Clin. Nutr..

[193] Smith G.I., Atherton P., Reeds D.N., Mohammed B.S., Rankin D., Rennie M.J., Mittendorfer B. (2011). Omega-3 polyunsaturated fatty acids augment the muscle protein anabolic response to hyperinsulinaemia-hyperaminoacidaemia in healthy young and middle-aged men and women. Clin. Sci..

[194] Baker E.J., Miles E.A., Burdge G.C., Yaqoob P., Calder P.C. (2016). Metabolism and functional effects of plant-derived omega-3 fatty acids in humans. Prog. Lipid Res..

[195] Smith G.I., Atherton P., Reeds D.N., Mohammed B.S., Rankin D., Rennie M.J., Mittendorfer B. (2011). Dietary omega-3 fatty acid supplementation increases the rate of muscle protein synthesis in older adults: A randomized controlled trial. Am. J. Clin. Nutr..

[196] Hussey H.J., Tisdale M.J. (1999). Effect of a cachectic factor on carbohydrate metabolism and attenuation by eicosapentaenoic acid. Br. J. Cancer.

[197] Wang H., Lai Y.J., Chan Y.L., Li T.L., Wu C.J. (2011). Epigallocatechin-3-gallate effectively attenuates skeletal muscle atrophy caused by cancer cachexia. Cancer Lett..

[198] Davis J.M., Murphy E.A., Carmichael M.D., Davis B. (2009). Quercetin increases brain and muscle mitochondrial biogenesis and exercise tolerance. Am. J. Physiol. Regul. Integr. Comp. Physiol..

[199] Alway S.E., McCrory J.L., Kearcher K., Vickers A., Frear B., Gilleland D.L., Bonner D.E., Thomas J.M., Donley D.A., Lively M.W. (2017). Resveratrol Enhances Exercise-Induced Cellular and Functional Adaptations of Skeletal Muscle in Older Men and Women. J. Gerontol. Ser. A Biol. Sci. Med. Sci..

[200] Davis J.M., Murphy E.A., Carmichael M.D. (2009). Effects of the dietary flavonoid quercetin upon performance and health. Curr. Sports Med. Rep..

[201] Aquila G., Re Cecconi A.D., Brault J.J., Corli O., Piccirillo R. (2020). Nutraceuticals and Exercise against Muscle Wasting during Cancer Cachexia. Cells.

[202] Antoun S., Raynard B. (2018). Muscle protein anabolism in advanced cancer patients: Response to protein and amino acids support, and to physical activity. Ann. Oncol..

[203] Kawamura I., Sato H., Ogoshi S., Blackburn G.L. (1985). Use of an intravenous branched chain amino acid enriched diet in the tumor-bearing rat. Jpn. J. Surg..

[204] Crosby L.E., Bistrian B.R., Ling P.R., Istfan N.W., Blackburn G.L., Hoffman S.B. (1988). Effects of branched chain amino acid-enriched total parenteral nutrition on amino acid utilization in rats bearing Yoshida sarcoma. Cancer Res..

[205] Eley H.L., Russell S.T., Tisdale M.J. (2007). Effect of branched-chain amino acids on muscle atrophy in cancer cachexia. Biochem. J..

[206] Penna F., Ballarò R., Beltrá M., De Lucia S., Costelli P. (2018). Modulating metabolism to improve cancer-induced muscle wasting. Oxid. Med. Cell. Longev..

[207] Girgis C.M., Clifton-Bligh R.J., Hamrick M.W., Holick M.F., Gunton J.E. (2013). The roles of vitamin D in skeletal muscle: Form, function, and metabolism. Endocr. Rev..

[208] Owens D.J., Sharples A.P., Polydorou I., Alwan N., Donovan T., Tang J., Fraser W.D., Cooper R.G., Morton J.P., Stewart C. (2015). A systems-based investigation into vitamin D and skeletal muscle repair, regeneration, and hypertrophy. Am. J. Physiol. Endocrinol. Metab..

[209] Von Hurst P.R., Stonehouse W., Coad J. (2010). Vitamin D supplementation reduces insulin resistance in South Asian women living in New Zealand who are insulin resistant and vitamin D deficient-a randomised, placebo-controlled trial. Br. J. Nutr..

[210] Lemieux P., John Weisnagel S., Caron A.Z., Julien A.S., Morisset A.S., Carreau A.M., Poirier J., Tchernof A., Robitaille J., Bergeron J. (2019). Effects of 6-month Vitamin D supplementation on insulin sensitivity and secretion: A randomised, placebo-controlled trial. Eur. J. Endocrinol..

[211] Mager D.R., Carroll M.W., Wine E., Siminoski K., MacDonald K., Kluthe C.L., Medvedev P., Chen M., Wu J., Turner J.M. (2018). Vitamin D status and risk for sarcopenia in youth with inflammatory bowel diseases. Eur. J. Clin. Nutr..

[212] Snijder M.B., Van Schoor N.M., Pluijm S.M.F., Van Dam R.M., Visser M., Lips P. (2006). Vitamin D Status in Relation to One-Year Risk of Recurrent Falling in Older Men and Women. J. Clin. Endocrinol. Metab..

[213] Garcia M., Seelaender M., Sotiropoulos A., Coletti D., Lancha A.H. (2019). Vitamin D, muscle recovery, sarcopenia, cachexia, and muscle atrophy. Nutrition.

[214] Dev R., Del Fabbro E., Schwartz G.G., Hui D., Palla S.L., Gutierrez N., Bruera E. (2011). Preliminary Report: Vitamin D Deficiency in Advanced Cancer Patients with Symptoms of Fatigue or Anorexia. Oncologist.

[215] Sustova H., De Feudis M., Reano S., Alves Teixeira M., Valle I., Zaggia I., Agosti E., Prodam F., Filigheddu N. (2019). Opposing effects of 25-hydroxy- and 1α,25-dihydroxy-vitamin D3 on pro-cachectic cytokine-and cancer conditioned medium-induced atrophy in C2C12 myotubes. Acta Physiol..

[216] Camperi A., Pin F., Costamagna D., Penna F., Menduina M.L., Aversa Z., Zimmers T., Verzaro R., Fittipaldi R., Caretti G. (2017). Vitamin D and VDR in cancer cachexia and muscle regeneration. Oncotarget.

[217] Ryan Z.C., Craig T.A., Wang X., Delmotte P., Salisbury J.L., Lanza I.R., Sieck G.C., Kumar R. (2018). 1α,25-dihydroxyvitamin D3 mitigates cancer cell mediated mitochondrial dysfunction in human skeletal muscle cells. Biochem. Biophys. Res. Commun..

[218] Padrão A.I., Figueira A.C.C., Faustino-Rocha A.I., Gama A., Loureiro M.M., Neuparth M.J., Moreira-Gonçalves D., Vitorino R., Amado F., Santos L.L. (2017). Long-term exercise training prevents mammary tumorigenesis-induced muscle wasting in rats through the regulation of TWEAK signalling. Acta Physiol..

[219] Battaglini C.L., Hackney A.C., Goodwin M.L. (2012). Cancer cachexia: Muscle physiology and exercise training. Cancers.

[220] Eschke R.C.K.R., Lampit A., Schenk A., Javelle F., Steindorf K., Diel P., Bloch W., Zimmer P. (2019). Impact of physical exercise on growth and progression of cancer in rodents-a systematic review and meta-analysis. Front. Oncol..

[221] Bacuau R.F.P., Belmonte M.A., Seelaender M.C.L., Costa Rosa L.F.B.P. (2000). Effect of a moderate intensity exercise training protocol on the metabolism of macrophages and lymphocytes of tumour-bearing rats. Cell Biochem. Funct..

[222] Bacurau A.V.N., Belmonte M.A., Navarro F., Moraes M.R., Pontes F.L., Pesquero J.L., Araújo R.C., Bacurau R.F.P. (2007). Effect of a high-intensity exercise training on the metabolism and function of macrophages and lymphocytes of walker 256 tumor-bearing rats. Exp. Biol. Med..

[223] Ranjbar K.I.A., Ballarò R., Bover Q., Pin F., Beltrà M., Penna F., Costelli P. (2019). Combined Exercise Training Positively Affects Muscle Wasting in Tumor-Bearing Mice. Med. Sci. Sports Exerc..

[224] Ballarò R., Beltrà M., De Lucia S., Pin F., Ranjbar K., Hulmi J.J., Costelli P., Penna F. (2019). Moderate exercise in mice improves cancer plus chemotherapy-induced muscle wasting and mitochondrial alterations. FASEB J..

[225] Aversa Z., Pin F., Lucia S., Penna F., Verzaro R., Fazi M., Colasante G., Tirone A., Fanelli F.R., Ramaccini C. (2016). Autophagy is induced in the skeletal muscle of cachectic cancer patients. Sci. Rep..

[226] He C., Bassik M.C., Moresi V., Sun K., Wei Y., Zou Z., An Z., Loh J., Fisher J., Sun Q. (2012). Exercise-induced BCL2-regulated autophagy is required for muscle glucose homeostasis. Nature.

[227] Narkar V.A., Downes M., Yu R.T., Embler E., Wang Y.X., Banayo E., Mihaylova M.M., Nelson M.C., Zou Y., Juguilon H. (2008). AMPK and PPARδ Agonists Are Exercise Mimetics. Cell.

[228] Handschin C. (2016). Caloric restriction and exercise “mimetics” Ready for prime time?. Pharmacol. Res..

[229] Penna F., Ballarò R., Martinez-Cristobal P., Sala D., Sebastian D., Busquets S., Muscaritoli M., Argilés J.M., Costelli P., Zorzano A. (2019). Autophagy Exacerbates Muscle Wasting in Cancer Cachexia and Impairs Mitochondrial Function. J. Mol. Biol..

[230] Montalvo R.N., Hardee J.P., Vanderveen B.N., Carson J.A. (2018). Resistance Exercise’s Ability to Reverse Cancer-Induced Anabolic Resistance. Exerc. Sport Sci. Rev..

[231] Lira F.S., Antunes B.D.M.M., Seelaender M., Neto J.C.R. (2015). The therapeutic potential of exercise to treat cachexia. Curr. Opin. Support. Palliat. Care.

[232] Puppa M.J., Murphy E.A., Fayad R., Hand G.A., Carson J.A. (2014). Cachectic skeletal muscle response to a novel bout of low-frequency stimulation. J. Appl. Physiol..

[233] Van der Ende M., Grefte S., Plas R., Meijerink J., Witkamp R.F., Keijer J., van Norren K. (2018). Mitochondrial dynamics in cancer-induced cachexia. Biochim. Biophys. Acta Rev. Cancer.

[234] Halle J.L., Counts B.R., Carson J.A. (2020). Exercise as a therapy for cancer-induced muscle wasting. Sport. Med. Health Sci..

[235] Carson J.A., Hardee J.P., VanderVeen B.N. (2016). The emerging role of skeletal muscle oxidative metabolism as a biological target and cellular regulator of cancer-induced muscle wasting. Semin. Cell Dev. Biol..

[236] Pin F., Busquets S., Toledo M., Camperi A., Lopez-Soriano F.J., Costelli P., Argilés J.M., Penna F. (2015). Combination of exercise training and erythropoietin prevents cancer-induced muscle alterations. Oncotarget.

[237] Wang X., Pickrell A.M., Zimmers T.A., Moraes C.T. (2012). Increase in muscle mitochondrial biogenesis does not prevent muscle loss but increased tumor size in a mouse model of acute cancer-induced cachexia. PLoS ONE.

[238] Ballarò R., Penna F., Ferraro E., Costelli P. (2019). Muscle mitochondria and oxidative metabolism as targets against cancer cachexia. J. Cancer Metastasis Treat..

[239] Kido K., Ato S., Yokokawa T., Makanae Y., Sato K., Fujita S. (2016). Acute resistance exercise-induced IGF1 expression and subsequent GLUT4 translocation. Physiol. Rep..

[240] Egan B., Zierath J.R. (2013). Exercise metabolism and the molecular regulation of skeletal muscle adaptation. Cell Metab..

[241] Honors M.A., Kinzig K.P. (2012). The role of insulin resistance in the development of muscle wasting during cancer cachexia. J. Cachexia Sarcopenia Muscle.

[242] Buffart L.M., Galvão D.A., Brug J., Chinapaw M.J.M., Newton R.U. (2014). Evidence-based physical activity guidelines for cancer survivors: Current guidelines, knowledge gaps and future research directions. Cancer Treat. Rev..

[243] Fuller J.T., Hartland M.C., Maloney L.T., Davison K. (2018). Therapeutic effects of aerobic and resistance exercises for cancer survivors: A systematic review of meta-analyses of clinical trials. Br. J. Sports Med..

[244] Mustian K.M., Sprod L.K., Janelsins M., Peppone L.J., Mohile S. (2012). Exercise Recommendations for Cancer-Related Fatigue, Cognitive Impairment, Sleep problems, Depression, Pain, Anxiety, and Physical Dysfunction: A Review. Oncol. Hematol. Rev..

[245] Bushman B.A. (2017). Complete Guide to Fitness & Health.

[246] Schmitz K.H., Courneya K.S., Matthews C., Demark-Wahnefried W., Galvão D.A., Pinto B.M., Irwin M.L., Wolin K.Y., Segal R.J., Lucia A. (2010). American College of Sports Medicine roundtable on exercise guidelines for cancer survivors. Med. Sci. Sports Exerc..

[247] Wolin K.Y., Schwartz A.L., Matthews C.E., Courneya K.S., Schmitz K.H. (2012). Implementing the exercise guidelines for cancer survivors. J. Support. Oncol..

[248] Penna F., Pin F., Ballarò R., Baccino F.M., Costelli P. (2016). Novel investigational drugs mimicking exercise for the treatment of cachexia. Expert Opin. Investig. Drugs.

[249] Gatta L., Vitiello L., Gorini S., Chiandotto S., Costelli P., Giammarioli A.M., Malorni W., Rosano G., Ferraro E. (2017). Modulating the metabolism by trimetazidine enhances myoblast differentiation and promotes myogenesis in cachectic tumor-bearing c26 mice. Oncotarget.

[250] De Lima E.A., Teixeira A.A.d.S., Biondo L.A., Diniz T.A., Silveira L.S., Coletti D., Rius S.B., Neto J.C.R. (2020). Exercise reduces the resumption of tumor growth and proteolytic pathways in the skeletal muscle of mice following chemotherapy. Cancers.

[251] Falcão-Tebas F., Bento-Santos A., Antônio Fidalgo M., De Almeida M.B., Dos Santos J.A., De Souza S.L., Manhães-De-Castro R., Leandro C.G. (2012). Maternal low-protein diet-induced delayed reflex ontogeny is attenuated by moderate physical training during gestation in rats. Br. J. Nutr..

[252] Fidalgo M., Falcão-Tebas F., Bento-Santos A., De Oliveira E., Nogueira-Neto J.F., De Moura E.G., Lisboa P.C., De Castro R.M., Leandro C.G. (2013). Programmed changes in the adult rat offspring caused by maternal protein restriction during gestation and lactation are attenuated by maternal moderate-low physical training. Br. J. Nutr..

[253] Bayol S.A., Simbi B.H., Stickland N.C. (2005). A maternal cafeteria diet during gestation and lactation promotes adiposity and impairs skeletal muscle development and metabolism in rat offspring at weaning. J. Physiol..

[254] Stanford K.I., Lee M.Y., Getchell K.M., So K., Hirshman M.F., Goodyear L.J. (2015). Exercise before and during pregnancy prevents the deleterious effects of maternal high-fat feeding on metabolic health of male offspring. Diabetes.

[255] Masuyama H., Hiramatsu Y. (2012). Effects of a high-fat diet exposure in utero on the metabolic syndrome-like phenomenon in mouse offspring through epigenetic changes in adipocytokine gene expression. Endocrinology.

[256] Fernandez-Twinn D.S., Hjort L., Novakovic B., Ozanne S.E., Saffery R. (2019). Intrauterine programming of obesity and type 2 diabetes. Diabetologia.

[257] Godfrey K.M., Reynolds R.M., Prescott S.L., Nyirenda M., Jaddoe V.W.V., Eriksson J.G., Broekman B.F.P. (2017). Influence of maternal obesity on the long-term health of offspring. Lancet Diabetes Endocrinol..

[258] Armitage J., Poston L., Taylor P. (2007). Developmental origins of obesity and the metabolic syndrome: The role of maternal obesity. Front. Horm. Res..

[259] Prats-Puig A., García-Retortillo S., Puig-Parnau M., Vasileva F., Font-Lladó R., Xargay-Torrent S., Carreras-Badosa G., Mas-Parés B., Bassols J., López-Bermejo A. (2020). DNA Methylation Reorganization of Skeletal Muscle-Specific Genes in Response to Gestational Obesity. Front. Physiol..

[260] Houshmand-Oeregaard A., Schrölkamp M., Kelstrup L., Hansen N.S., Hjort L., Thuesen A.C.B., Broholm C., Mathiesen E.R., Clausen T.D., Vaag A. (2018). Increased expression of microRNA-15a and microRNA-15b in skeletal muscle from adult offspring of women with diabetes in pregnancy. Hum. Mol. Genet..

[261] Laker R.C., Wlodek M.E., Connelly J., Yan Z. (2013). Epigenetic origins of metabolic disease: The impact of the maternal condition to the offspring epigenome and later health consequences. Food Sci. Hum. Wellness.

[262] Simar D., Chen H., Lambert K., Mercier J., Morris M.J. (2012). Interaction between maternal obesity and post-natal over-nutrition on skeletal muscle metabolism. Nutr. Metab. Cardiovasc. Dis..

[263] Beleza J., Stevanović-Silva J., Coxito P., Costa R.C., Ascensão A., Torrella J.R., Magalhães J. (2021). Building-up fit muscles for the future: Transgenerational programming of skeletal muscle through physical exercise. Eur. J. Clin. Investig..

[264] Bayol S.A., MacHaria R., Farrington S.J., Simbi B.H., Stickland N.C. (2009). Evidence that a maternal “junk food” diet during pregnancy and lactation can reduce muscle force in offspring. Eur. J. Nutr..

[265] Walter I., Klaus S. (2014). Maternal high-fat diet consumption impairs exercise performance in offspring. J. Nutr. Sci..

[266] Pileggi C.A., Hedges C.P., Segovia S.A., Markworth J.F., Durainayagam B.R., Gray C., Zhang X.D., Barnett M.P.G., Vickers M.H., Hickey A.J.R. (2016). Maternal high fat diet alters skeletal muscle mitochondrial catalytic activity in adult male rat offspring. Front. Physiol..

[267] Latouche C., Heywood S.E., Henry S.L., Ziemann M., Lazarus R., El-Osta A., Armitage J.A., Kingwell B.A. (2014). Maternal overnutrition programs changes in the expression of skeletal muscle genes that are associated with insulin resistance and defects of oxidative phosphorylation in adult male rat offspring. J. Nutr..

[268] Bodden C., Hannan A.J., Reichelt A.C. (2020). Diet-Induced Modification of the Sperm Epigenome Programs Metabolism and Behavior. Trends Endocrinol. Metab..

[269] Su L., Patti M.E. (2019). Paternal Nongenetic Intergenerational Transmission of Metabolic Disease Risk. Curr. Diabetes Rep..

[270] De Castro Barbosa T., Ingerslev L.R., Alm P.S., Versteyhe S., Massart J., Rasmussen M., Donkin I., Sjögren R., Mudry J.M., Vetterli L. (2016). High-fat diet reprograms the epigenome of rat spermatozoa and transgenerationally affects metabolism of the offspring. Mol. Metab..

[271] Kusuyama J., Alves-Wagner A.B., Makarewicz N.S., Goodyear L.J. (2020). Effects of maternal and paternal exercise on offspring metabolism. Nat. Metab..

[272] Raipuria M., Bahari H., Morris M.J. (2015). Effects of maternal diet and exercise during pregnancy on glucose metabolism in skeletal muscle and fat of weanling rats. PLoS ONE.

[273] Harris J.E., Baer L.A., Stanford K.I. (2018). Maternal Exercise Improves the Metabolic Health of Adult Offspring. Trends Endocrinol. Metab..

[274] Laker R.C., Lillard T.S., Okutsu M., Zhang M., Hoehn K.L., Connelly J.J., Yan Z. (2014). Exercise prevents maternal high-fat diet-induced hypermethylation of the Pgc-1α gene and age-dependent metabolic dysfunction in the offspring. Diabetes.

[275] Kasch J., Kanzleiter I., Saussenthaler S., Schürmann A., Keijer J., van Schothorst E., Klaus S., Schumann S. (2018). Insulin sensitivity linked skeletal muscle Nr4a1 DNA methylation is programmed by the maternal diet and modulated by voluntary exercise in mice. J. Nutr. Biochem..

[276] Falcão-Tebas F., Marin E.C., Kuang J., Bishop D.J., McConell G.K. (2020). Maternal exercise attenuates the lower skeletal muscle glucose uptake and insulin secretion caused by paternal obesity in female adult rat offspring. J. Physiol..

[277] McPherson N.O., Owens J.A., Fullston T., Lane M. (2015). Preconception diet or exercise intervention in obese fathers normalizes sperm microRNA profile and metabolic syndrome in female offspring. Am. J. Physiol. Endocrinol. Metab..

[278] Hargreaves M., Spriet L.L. (2020). Skeletal muscle energy metabolism during exercise. Nat. Metab..

[279] Lundby C., Jacobs R.A. (2016). Adaptations of skeletal muscle mitochondria to exercise training. Exp. Physiol..

[280] Hoppeler H. (2016). Molecular networks in skeletal muscle plasticity. J. Exp. Biol..

[281] McGee S.L., Fairlie E., Garnham A.P., Hargreaves M. (2009). Exercise-induced histone modifications in human skeletal muscle. J. Physiol..

[282] Ultimo S., Zauli G., Martelli A.M., Vitale M., McCubrey J.A., Capitani S., Neri L.M. (2018). Influence of physical exercise on microRNAs in skeletal muscle regeneration, aging and diseases. Oncotarget.

[283] Murphy R.M., Watt M.J., Febbraio M.A. (2020). Metabolic communication during exercise. Nat. Metab..

[284] Stanford K.I., Rasmussen M., Baer L.A., Lehnig A.C., Rowland L.A., White J.D., So K., De Sousa-Coelho A.L., Hirshman M.F., Patti M.E. (2018). Paternal exercise improves glucose metabolism in adult offspring. Diabetes.

[285] Ferrari N., Bae-Gartz I., Bauer C., Janoschek R., Koxholt I., Mahabir E., Appel S., Alejandre Alcazar M.A., Grossmann N., Vohlen C. (2018). Exercise during pregnancy and its impact on mothers and offspring in humans and mice. J. Dev. Orig. Health Dis..

[286] Koletzko B., Cremer M., Flothkötter M., Graf C., Hauner H., Hellmers C., Kersting M., Krawinkel M., Przyrembel H., Röbl-Mathieu M. (2018). Diet and Lifestyle before and during Pregnancy—Practical Recommendations of the Germany-wide Healthy Start—Young Family Network. Geburtshilfe Frauenheilkd..

[287] McMillan A.G., May L.E., Gaines G.G., Isler C., Kuehn D. (2019). Effects of Aerobic Exercise during Pregnancy on 1-Month Infant Neuromotor Skills. Med. Sci. Sports Exerc..

[288] Mourtakos S.P., Tambalis K.D., Panagiotakos D.B., Antonogeorgos G., Arnaoutis G., Karteroliotis K., Sidossis L.S. (2015). Maternal lifestyle characteristics during pregnancy, and the risk of obesity in the offspring: A study of 5,125 children. BMC Pregnancy Childbirth.

[289] Parastesh M., Heidarianpour A., Sadegh M. (2019). Investigating the effects of endurance, resistance and combined training on reproductive hormones and sperm parameters of streptozotocin–nicotinamide diabetic male rats. J. Diabetes Metab. Disord..

[290] Sun B., Messerlian C., Sun Z.H., Duan P., Chen H.G., Chen Y.J., Wang P., Wang L., Meng T.Q., Wang Q. (2019). Physical activity and sedentary time in relation to semen quality in healthy men screened as potential sperm donors. Hum. Reprod..

[291] Guth L.M., Ludlow A.T., Witkowski S., Marshall M.R., Lima L.C.J., Venezia A.C., Xiao T., Ting Lee M.L., Spangenburg E.E., Roth S.M. (2013). Sex-specific effects of exercise ancestry on metabolic, morphological and gene expression phenotypes in multiple generations of mouse offspring. Exp. Physiol..

[292] Murashov A.K., Pak E.S., Koury M., Ajmera A., Jeyakumar M., Parker M., Williams O., Ding J., Walters D., Neufer P.D. (2016). Paternal long-term exercise programs offspring for low energy expenditure and increased risk for obesity in mice. FASEB J..

[293] Cedola A., Mastrogiacomo M., Lagomarsino S., Cancedda R., Giannini C., Guagliardi A., Ladisa M., Burghammer M., Rustichelli F., Komlev V. (2007). Orientation of mineral crystals by collagen fibers during in vivo bone engineering: An X-ray diffraction imaging study. Spectrochim. Acta Part B At. Spectrosc..

